# Astrocytic control of extracellular GABA drives circadian timekeeping in the suprachiasmatic nucleus

**DOI:** 10.1073/pnas.2301330120

**Published:** 2023-05-15

**Authors:** Andrew P. Patton, Emma L. Morris, David McManus, Huan Wang, Yulong Li, Jason W. Chin, Michael H. Hastings

**Affiliations:** ^a^Neurobiology Division, Medical Research Council Laboratory of Molecular Biology, Cambridge CB2 0QH, United Kingdom; ^b^State Key Laboratory of Membrane Biology, Peking University, School of Life Sciences, 100871 Beijing, China; ^c^PNAC Division, Medical Research Council Laboratory of Molecular Biology, Cambridge CB2 0QH, United Kingdom

**Keywords:** circadian rhythms, suprachiasmatic nucleus, GABA, astrocytes, GABA transporters

## Abstract

The hypothalamic suprachiasmatic nucleus (SCN) is the master mammalian circadian clock. Its cell-autonomous timing mechanism, a transcriptional/translational feedback loop (TTFL), drives daily peaks of neuronal electrical activity, which in turn control circadian behavior. Intercellular signals, mediated by neuropeptides, synchronize and amplify TTFL and electrical rhythms across the circuit. SCN neurons are GABAergic, but the role of GABA in circuit-level timekeeping is unclear. How can a GABAergic circuit sustain circadian cycles of electrical activity, when such increased neuronal firing should become inhibitory to the network? To explore this paradox, we show that SCN slices expressing the GABA sensor iGABASnFR demonstrate a circadian oscillation of extracellular GABA ([GABA]_e_) that, counterintuitively, runs in antiphase to neuronal activity, with a prolonged peak in circadian night and a pronounced trough in circadian day. Resolving this unexpected relationship, we found that [GABA]_e_ is regulated by GABA transporters (GATs), with uptake peaking during circadian day, hence the daytime trough and nighttime peak. This uptake is mediated by the astrocytically expressed transporter GAT3 (*Slc6a11*), expression of which is circadian-regulated, being elevated in daytime. Clearance of [GABA]_e_ in circadian day facilitates neuronal firing and is necessary for circadian release of the neuropeptide vasoactive intestinal peptide, a critical regulator of TTFL and circuit-level rhythmicity. Finally, we show that genetic complementation of the astrocytic TTFL alone, in otherwise clockless SCN, is sufficient to drive [GABA]_e_ rhythms and control network timekeeping. Thus, astrocytic clocks maintain the SCN circadian clockwork by temporally controlling GABAergic inhibition of SCN neurons.

## Significance

Circadian clocks drive daily physiological rhythms that adapt us to day and night. Their disruption by societal pressures (shift-work) or disease (neurodegeneration) carries severe economic and health consequences. The suprachiasmatic nucleus (SCN) is the principal brain clock, coordinating rhythms across the body. It consists of interconnected neurons that use the inhibitory neurotransmitter GABA, alongside astrocyte supporting cells. Using microscopic imaging, pharmacology, and molecular genetic approaches, we show that astrocytes sustain SCN timekeeping by controlling a daily rhythm of GABA levels. Their reduction during the day, by enhanced clearance, provides a circadian window for neuronal activity. This resolves a long-standing paradox of how an exclusively inhibitory network sustains neuronal activity and highlights the critical importance of astrocyte-to-neuron signaling in the clock.

The suprachiasmatic nucleus (SCN) of the hypothalamus is the master circadian (~daily) clock in mammals. It coordinates the timing of distributed peripheral tissue clocks, thereby controlling adaptive behavioral and physiological rhythms. It is synchronized to environmental time by innervation from the retinohypothalamic tract (RHT) (1), but the RHT is dispensable for robust SCN timekeeping. When isolated ex vivo, the SCN sustains precise circadian rhythms of molecular, electrical, and metabolic activity.

At the cellular level, timekeeping pivots around a core transcriptional/translational feedback loop (TTFL), whereby the transcription factors CLOCK and BMAL1 drive expression of *Period* and *Cryptochrome* genes, and their encoded proteins, PER1/2, and CRY1/2, subsequently repress that transactivation. This generates self-sustaining cycles of TTFL activity and its dependent transcriptional outputs (2), including ion channels (3). These in turn direct daily rhythms in electrical excitability of SCN neurons (4), peaking in circadian day, with quiescence during circadian night (4–6). Cell-autonomous timekeeping is augmented by reciprocal circuit-level neuron-to-glia signaling that determines emergent properties of the SCN network: precise high-amplitude oscillation, tightly defined ensemble period and phase, and spatiotemporally complex cellular synchrony (7, 8). These network-level mechanisms are so powerful that they sustain robust rhythms with periods ranging between 16 and 42 h in various genetic/pharmacological contexts (9). Neuronal electrical rhythms are integral to this synchronization through appropriately timed neurotransmitter/neuropeptide release: Pharmacological blockade of neuronal firing (10) and synaptic vesicle release (11) desynchronize and damp network oscillations.

SCN neurons are neurochemically heterogeneous due to their complex expression of a wide array of neuropeptides and receptors (12–16) but are also homogeneous insofar as almost all utilize the inhibitory small neurotransmitter γ-aminobutyric acid (GABA) (17). GABAergic neurotransmission is mediated via ionotropic GABA_A_ or metabotropic GABA_B_-receptors, and both subtypes are expressed within the SCN (17) together with GABA transporters (GATs) (18). Nevertheless, little is known about the role of GABA in steady-state network timekeeping (17, 19). It has been considered as a counterbalance to neuropeptidergic signaling (20), a synchronizing factor (21), a modulator of encoded phase (17), and a network adaptor to seasonal changes in day length (22–24). Pharmacological and genetic disruption of GABAergic signaling does not, however, disrupt TTFL oscillations and circadian firing of SCN neurons ex vivo (9, 25, 26). This suggests that although GABA is important in vivo as an SCN output acting on distal targets (via GABAergic synaptic terminals) (26, 27), it is largely dispensable at the network level. Indeed, how could a circuit that exclusively uses GABA as a neurotransmitter alongside neuropeptides sustain circadian electrical activity, when increased neuronal firing would inhibit the network via this GABAergic output?

To reconcile this paradox and identify a role for GABA within the SCN network, we first monitored real-time extracellular GABA dynamics ([GABA]_e_) in free-running SCN explants. This revealed a counterintuitive daily bulk [GABA]_e_ flux, peaking at night in antiphase to (GABAergic) neuronal activity. This rhythm is not, however, driven by circadian variation in neuronal activity, but is rather driven by daytime GABA uptake via GATs, GAT1 (*Slc6a1*, mGAT1), and GAT3 (*Slc6a11*, mGAT4). Furthermore, this rhythmic uptake is controlled by the cell-autonomous circadian clock of astrocytes. This reveals a GABAergic network mechanism whereby active daytime uptake is permissive to neuronal activation and neuropeptide release during circadian day. Thus, the SCN circuit has devolved GABAergic control of its neurons to its astrocytes and their cell-autonomous clock to sustain and encode circadian time.

## Results

### Extracellular GABA is Rhythmic in the SCN and Peaks during Circadian Night.

**Fig. 1.**
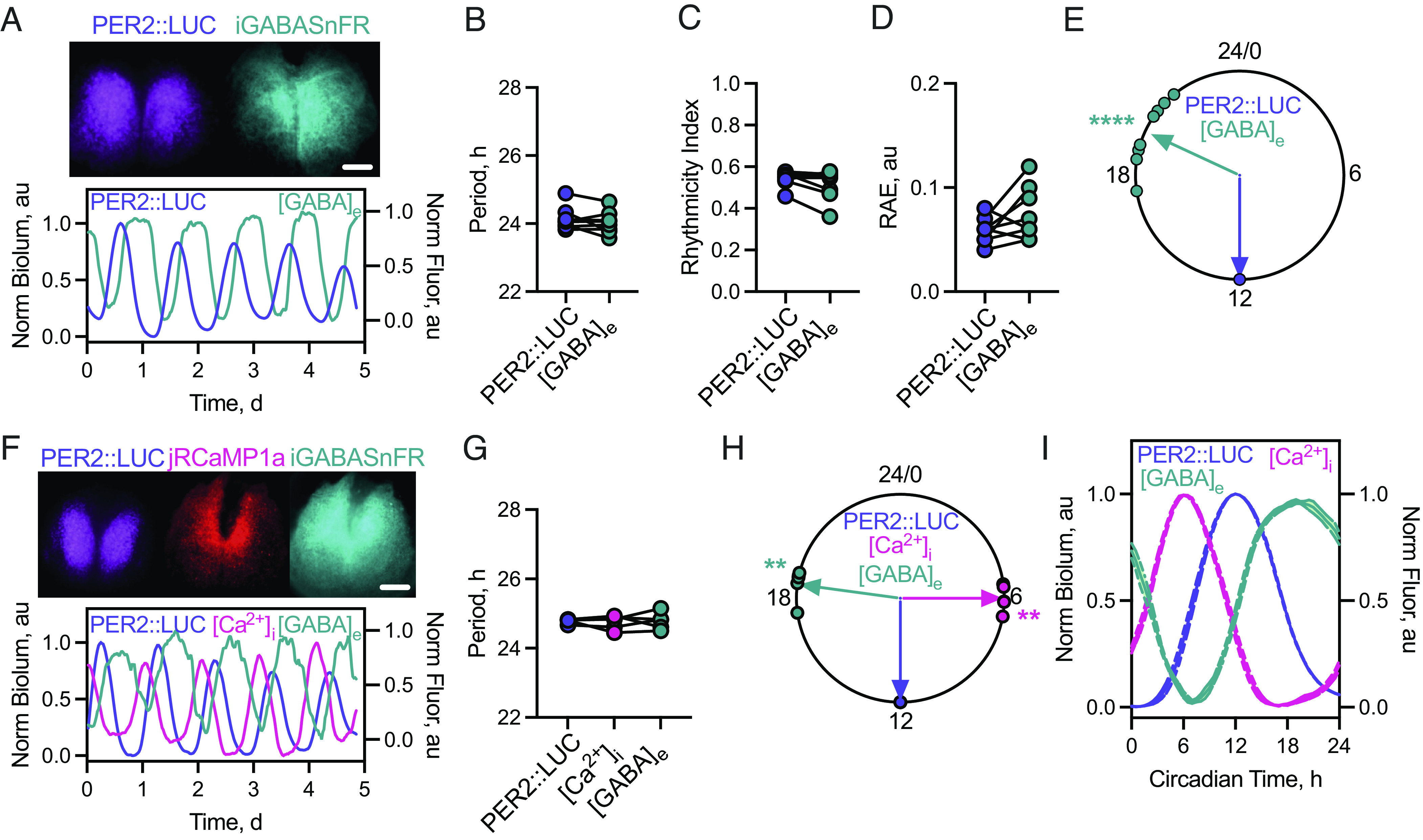
iGABASnFR reports robust oscillations in [GABA]_e_ in antiphase to SCN neuronal activity. (*A*, *Upper*) Average Z-projections of PER2::LUC (*Left*) bioluminescence and *Syn.*iGABASnFR (*Right*) fluorescence in an ex vivo SCN slice. (*A*, *Lower*) Example normalized PER2::LUC bioluminescence and *Syn.*iGABASnFR fluorescence ([GABA]_e_). (*B*–*D*) Histograms showing aggregate statistics for PER2::LUC and [GABA]_e_ rhythms paired by SCN slice for: period (*B*), rhythmicity index (*C*), and RAE (*D*). (*E*) Circular Rayleigh plot showing the relative phasing of peak [GABA]_e_ aligned on a slice-by-slice basis to peak PER2::LUC. (*F*, *Upper*) Average Z-projections of PER2::LUC (*Left*) bioluminescence, *Syn*.jRCaMP1a (*Middle*), and *Syn*.iGABASnFR (*Right*) fluorescence. (*Lower*) Example trace showing normalized multiplexed PER2::LUC, *Syn.*jRCaMP1a ([Ca^2+^]_i_), and *Syn.*iGABASnFR ([GABA]_e_). (*G*) Histogram showing aggregate period for PER2::LUC, [Ca^2+^]_i_ and [GABA]_e_ rhythms paired by SCN slice. (*H*) Circular Rayleigh plot showing the relative phasing of the peak in [Ca^2+^]_i_ and [GABA]_e_ aligned to peak PER2::LUC. (*I*) Normalized single cycles of PER2::LUC, [Ca^2+^]_i_ and [GABA]_e_ rhythms aligned to peak PER2::LUC. In all plots, points represent SCN with paired measures joined. Lines/shading represent mean ± SEM. For *B*–*E* N = 8, *G*–*I* N = 4. (Scale bar, 250 µm.)

To determine the circadian dynamics of [GABA]_e_, we transduced SCN explants with adeno-associated viral vectors (AAVs) encoding the fluorescent GABA reporter iGABASnFR (28) controlled by the neuronal *synapsin* (*Syn*) promoter. Appropriate membrane-targeted fluorescence (*SI Appendix*, Fig. S1) was observed across the explant 1-wk post-transduction (Fig. 1*A*). Fluorescence was recorded for ~5 d in tandem with a bioluminescent TTFL reporter, PER2::Luciferase (PER2::LUC), which provided a circadian phase reference: PER2::LUC peaks at circadian time (CT)12, the start of circadian night (Fig. 1*A*). Imaging revealed rhythms in [GABA]_e_ (Movie S1) with a robust waveform consisting of a broad, flat peak and a sharp trough. The circadian properties of the [GABA]_e_ rhythm were comparable with those of PER2::LUC, with identical periods (Fig. 1*B*, period: PER2::LUC 24.14 ± 0.12 h vs. [GABA]_e_ 24.03 ± 0.12 h, paired two-tailed *t* test t(7) = 1.23, *P* = 0.26), rhythm quality (Fig. 1*C*, rhythmicity index (RI): PER2::LUC 0.54 ± 0.01 vs. [GABA]_e_ 0.52 ± 0.03, paired two-tailed *t* test t(7) = 1.45, *P* = 0.19), and precision (Fig. 1*D*, relative amplitude error (RAE): PER2::LUC 0.06 ± 0.01 vs. [GABA]_e_ 0.08 ± 0.01, paired two-tailed *t* test t(7) = 1.80, *P* = 0.12). Intriguingly, however, [GABA]_e_ peaked during circadian night in all slices (CT19.7 ± 0.48, Rayleigh test *P* < 0.0001, *R* = 0.95) (Fig. 1*E*), a counterintuitive finding given that the exclusively GABAergic SCN neurons are electrically active (and releasing synaptic GABA) in circadian daytime (~CT06).

Due to this unexpected observation, we then coregistered [GABA]_e_ explicitly with neuronal activity, reflected by intracellular calcium levels ([Ca^2+^]_i_). PER2::LUC SCN explants were cotransduced with *Syn.*iGABASnFR and *Syn.*jRCaMP1a. This, again, revealed robust rhythms of [GABA]_e_ alongside circadian oscillations of neuronal [Ca^2+^]_i_ (Fig. 1*F*). All three circadian markers had identical periods (Fig. 1*G*, PER2::LUC 24.79 ± 0.03 h vs. [Ca^2+^]_i_ 24.74 ± 0.09 h vs. [GABA]_e_ 24.78 ± 0.11 h, repeated-measures one-way ANOVA, F(2, 8) = 0.11, *P* = 0.90). As anticipated, [Ca^2+^]_i_ peaked in the day (CT5.99 ± 0.22) whereas in the same slices, [GABA]_e_ again peaked in circadian night (CT18.51 ± 0.27 h) and significantly delayed relative to [Ca^2+^]_i_ (paired two-tailed *t* test, t(4) = 34.51, *P* < 0.0001) across all slices tested (Rayleigh test: [Ca^2+^]_i_ *P* = 0.001, R = 0.99; [GABA]_e_ *P* = 0.001, R = 0.99) (Fig. 1*H*). Notably, average, single-cycle alignment of [Ca^2+^]_i_ and [GABA]_e_ rhythm waveforms mirrored one another: [GABA]_e_ dynamics appearing as an inversion of those of neuronal [Ca^2+^]_i_ (Fig. 1*I*). In SCN explants, therefore, the circadian rhythm of [GABA]_e_ sits in antiphase to neuronal rhythmicity, peaking in circadian night.

### Reduced GABAergic Tone during the Day Facilitates Network Timekeeping.

**Fig. 2.**
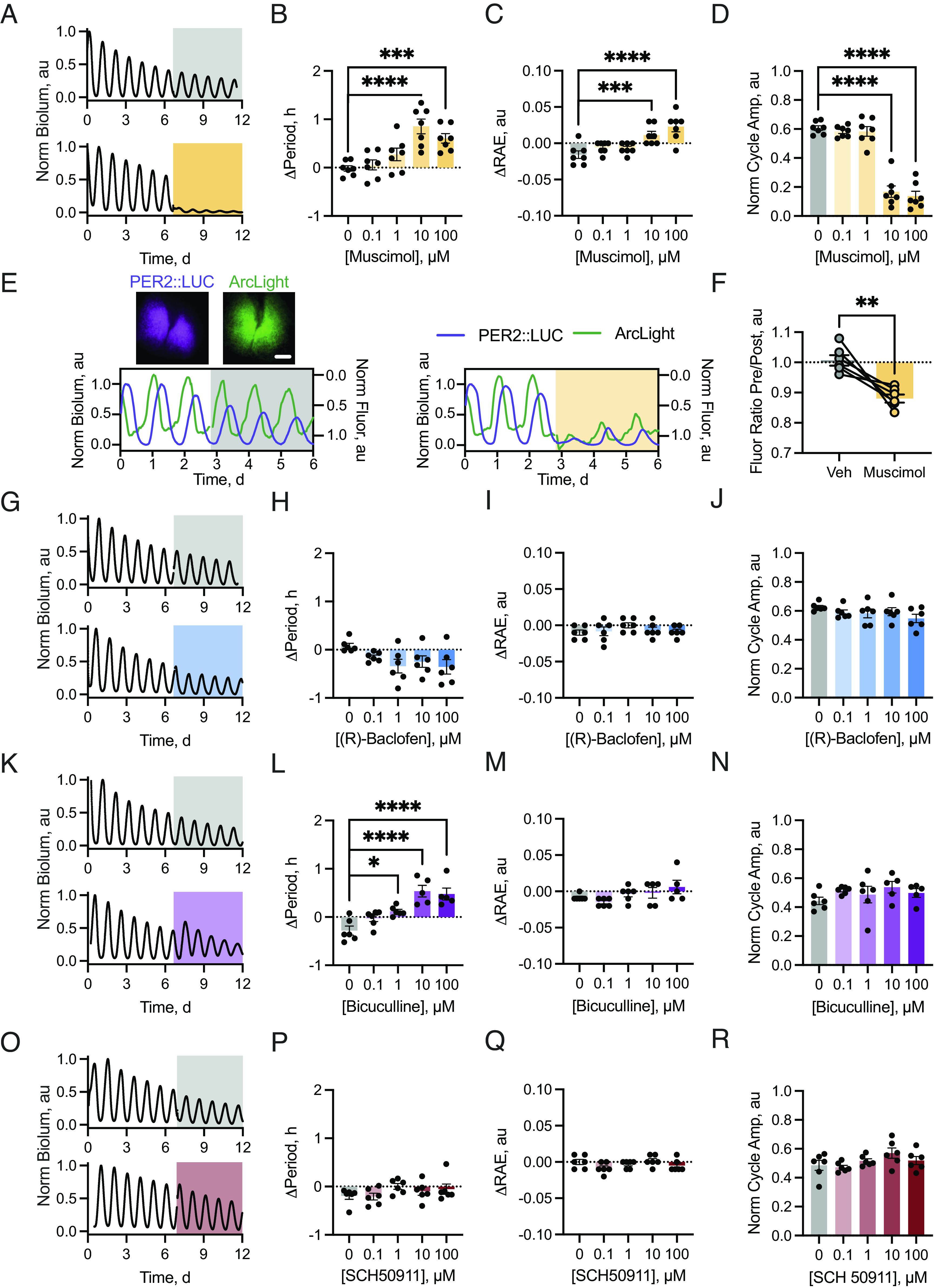
Chronic activation or inactivation of GABA_A_ receptors alters SCN network properties. (*A*) PER2::LUC bioluminescence from SCN treated with vehicle (*Upper*, gray) or 100 µM muscimol (*Lower*, orange). (*B*–*D*) Histogram showing the change in period (*B*), RAE (*C*) and normalized amplitude (*D*) between baseline and treatment intervals vs. muscimol concentration. (*E*, *Upper*) Average Z-projections of PER2::LUC (*Left*) bioluminescence and *Syn.*ArcLight (*Right*) fluorescence in an ex vivo SCN slice. (*E*, *Lower*) Example normalized PER2::LUC bioluminescence and *Syn-*ArcLight fluorescence from slices treated with vehicle (*Left*, gray shading) or 10 µM muscimol (*Right*, orange shading). Note: ArcLight recordings are inverted so that depolarization and hyperpolarization are represented by upward and downward inflections, respectively (29). (*F*) Relative shifts in total fluorescence expressed as a ratio of the changes over the last 24 h preceding and the first 24 h of treatment for vehicle (gray) and 10 µM muscimol treatment (orange). (*G*) PER2::LUC bioluminescence from SCN treated with vehicle (*Upper*, gray) or 100 µM (R)-baclofen (*Lower*, blue). (*H*–*J*) Histogram showing the change in period (*H*), RAE (*I*), and normalized amplitude (*J*) between baseline and treatment intervals vs. (R)-baclofen concentration. (*K*) PER2::LUC bioluminescence from SCN treated with vehicle (*Upper*, gray) or 100 µM (+)-bicuculline (*Lower*, purple). (*L*–*N*) Histogram showing the change in period (*L*), RAE (*M*), and normalized amplitude (*N*) between baseline and treatment intervals vs. (+)-bicuculline concentration. (*O*) PER2::LUC bioluminescence from SCN treated with vehicle (*Upper*, gray) or 100 µM SCH50911 (*Lower*, maroon). (*P*–*R*) Histogram showing the change in period (*P*), RAE (*Q*), and normalized amplitude (*R*) between baseline and treatment intervals vs. SCH50911 concentration. In all plots, points represent SCN (N ≥ 5 for all concentrations) and bars represent mean ± SEM. In *F*, joined points represent paired measures. (Scale bar, 250 µm.)

To explore the functional consequences of the [GABA]_e_ rhythm, we artificially clamped GABAergic tone into a chronic “high” condition, mimicking nighttime, by using the GABA_A_- or GABA_B_-receptor-specific agonists muscimol or (R)-baclofen, respectively. Chronic activation of GABA_A_-receptors by muscimol (Fig. 2*A*) dose-dependently lengthened circadian period (Fig. 2*B*, one-way ANOVA F(4, 24) = 13.91, *P* < 0.0001) and reduced both precision (RAE) (Fig. 2*C*, one-way ANOVA F(4, 24) = 12.21, *P* < 0.0001) and amplitude (Fig. 2*D*, one-way ANOVA F(4, 24) = 140.8, *P* < 0.0001) without any permanent effect on the oscillation, which was restored following washout (*SI Appendix*, Fig. S2*A*). This reduction in amplitude was associated with a marked suppression of both the level of the circadian trough (*SI Appendix*, Fig. S3*A*, one-way ANOVA F(4, 24) = 32.52, *P* < 0.0001) and peak (*SI Appendix*, Fig. S3*A*, one-way ANOVA F(4, 24) = 124.3, *P* < 0.0001), consistent with an electrically suppressed SCN (4). This electrically suppressive action was confirmed through membrane voltage recordings of the genetically encoded voltage indicator ArcLight (29) (Fig. 2*E*), which showed network hyperpolarization during 10 µM muscimol treatment compared to vehicle (Fig. 2*F*, vehicle vs. muscimol pre/post ratio: 1.01 ± 0.02 au vs. 0.88 ± 0.01 au, paired two-tailed *t* test t(6) = 4.305, *P* = 0.008). Intriguingly, the trough of PER2::LUC appeared more sensitive to muscimol at lower concentrations than did the peak (*SI Appendix*, Fig. S3*A*). In contrast, chronic activation of GABA_B_ receptors by (R)-baclofen (Fig. 2*G*) did not alter TTFL period (Fig. 2*H*, one-way ANOVA F(4, 20) = 2.39, *P* = 0.09), precision (Fig. 2*I*, one-way ANOVA F(4, 20) = 1.647, *P* = 0.20), or amplitude (Fig. 2*J*, one-way ANOVA F(4, 20) = 1.54, *P* = 0.23). Although there was no effect on overall amplitude, chronic activation of GABA_B_-receptors slightly suppressed the trough (*SI Appendix*, Fig. S3*B*, one-way ANOVA F(4, 20) = 3.252, *P* = 0.0330) without affecting the peak (*SI Appendix*, Fig. S3*B*, one-way ANOVA F(4, 20) = 2.50, *P* = 0.07). Thus, although sustained activation of GABA_A_-receptors compromised the TTFL, the TTFL was not affected by sustained activation of GABA_B_-receptors.

To complement these observations on high GABA tone, we then clamped GABAergic signaling into a chronic “low” condition to mimic daytime by using the GABA_A_- or GABA_B_-receptor specific antagonists (+)-bicuculline or SCH50911, respectively. Blockade of GABA_A_-receptors with (+)-bicuculline (Fig. 2*K*) slightly lengthened period at high doses (Fig. 2*L*, one-way ANOVA F(4, 23) = 14.16, *P* < 0.0001) without altering precision (Fig. 2*M*, one-way ANOVA F(4, 23) = 2.15, *P* = 0.11) or amplitude (Fig. 2*N*, one-way ANOVA F(4, 23) = 0.96, *P* = 0.45) and without any permanent effects on the ongoing oscillation, which continued following washout (*SI Appendix*, Fig. S2*B*). Despite the lack of effect of (+)-bicuculline on the overall cycle amplitude, there was a dose-dependent increase in the level of both the circadian trough (*SI Appendix*, Fig. S3*C*, one-way ANOVA F(4, 23) = 3.79, *P* = 0.02) and peak (*SI Appendix*, Fig. S3*C*, one-way ANOVA F(4, 23) = 8.65, *P* = 0.0002), consistent with increased electrical excitability feeding into the TTFL (4). In contrast, increasing doses of the GABA_B_-receptor antagonist SCH50911 (Fig. 2*O*) did not alter period (Fig. 2*P*, one-way ANOVA F(4, 25) = 1.32, *P* = 0.29), precision (Fig. 2*Q*, one-way ANOVA F(4, 25) = 1.64, *P* = 0.20), or amplitude (Fig. 2*R*, one-way ANOVA F(4, 25) = 1.99, *P* = 0.13). Equally, SCH50911 had no effect on the trough (*SI Appendix*, Fig. S3*D*, one-way ANOVA F(4, 25) = 1.62, *P* = 0.20) or peak (*SI Appendix*, Fig. S3*D*, one-way ANOVA F(4, 25) = 1.35, *P* = 0.28). Thus, loss of GABA_A_-signaling affects TTFL period and range of operation, whereas loss of GABA_B_ does not. Taken together, the data from agonists and antagonists demonstrate that GABAergic signaling acts via the GABA_A_- but not GABA_B_-receptors to suppress TTFL function in the SCN. This suggests that the low-level of GABA during circadian day facilitates daytime neuronal activity, which in turn boosts SCN network function (4).

### GABA Transporters Facilitate Ongoing SCN Rhythmicity.

**Fig. 3.**
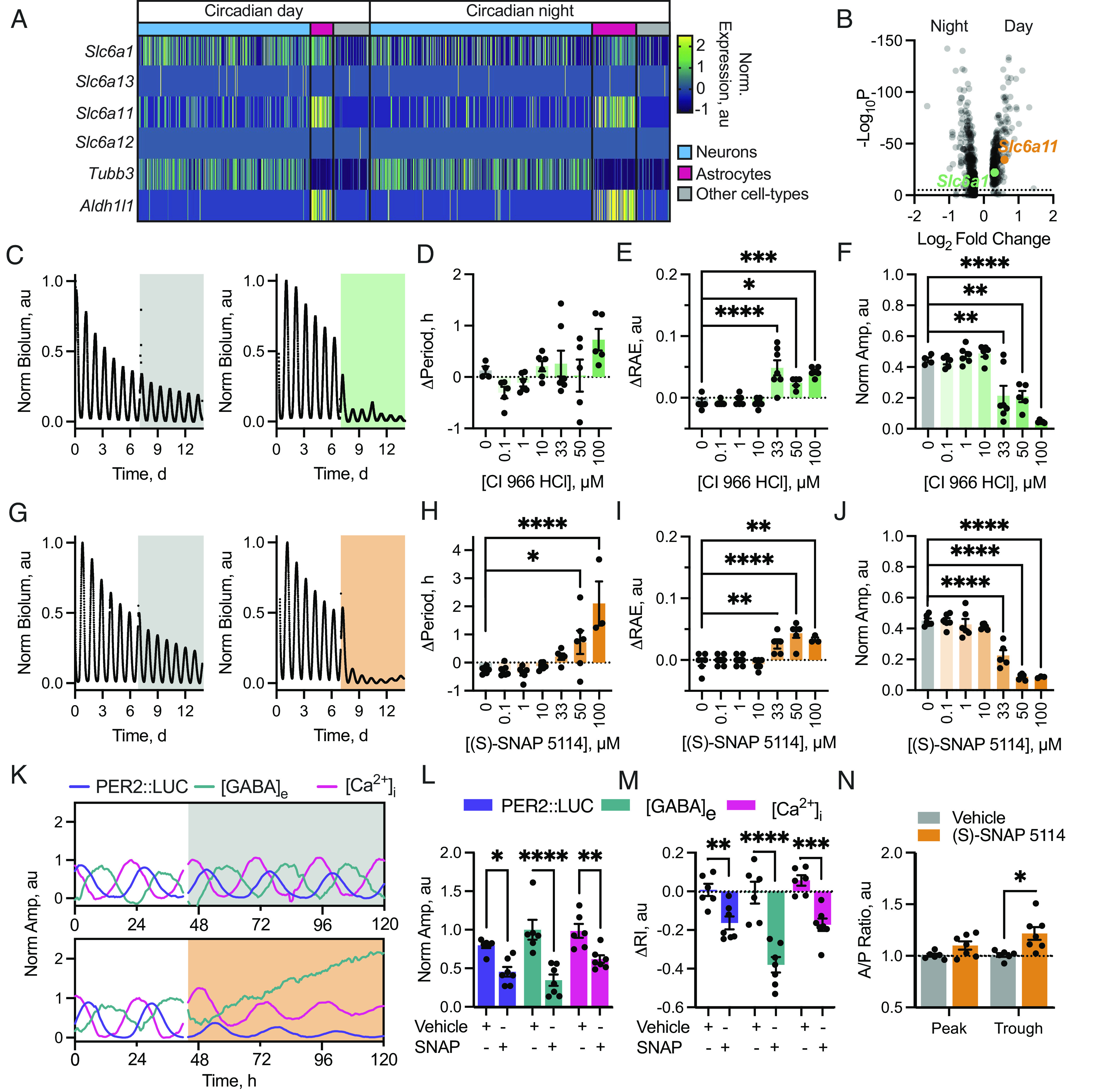
Inhibition of GATs disrupts circadian timekeeping and compromises [GABA]_e_ dynamics. (*A*) Heatmap of normalized expression levels of *Slc6a1* (GAT1, mGAT1), *Slc6a13* (GAT2, mGAT3), *Slc6a11* (GAT3, mGAT4), and *Slc6a12* (BGT1, mGAT2) alongside the neuronal *Tubb3* and astrocytic *Aldh1l1* markers. Cells types are indicated by coloured bars: neurons (blue), astrocytes (magenta), and all others (gray). Time of day at which cells were harvested is indicated. Reanalyzed from ref. (13, 30). (*B*) Volcano plot of differential day–night gene expression in astrocytes. Horizontal line indicates the significance cutoff. *Slc6a1* (GAT1, green) and *Slc6a11* (GAT3, orange) are significantly up-regulated in daytime. (*C*) PER2::LUC trace from SCN treated with vehicle (*Left*, gray) or 50 µM CI 966 HCl (*Right*, green). (*D*–*F*) Histogram showing change in period (*D*), RAE (*E*), and normalized amplitude (*F*) between baseline and treatment intervals vs. CI 966 HCl concentration. (*G*) PER2::LUC from vehicle (*Left*, gray) or 50 µM (S)-SNAP 5114 (*Right*, orange) treated slices. (*H*–*J*) Histogram showing the change in period (*H*), RAE (*I*) and normalized amplitude (*J*) between baseline and treatment intervals vs. (S)-SNAP 5114 concentration. (*K*) Example PER2::LUC, [GABA]_e_ and neuronal [Ca^2+^]_i_ traces from vehicle (*Left*, gray) or 50 µM (S)-SNAP 5114 (SNAP) (*Right*, orange) treated slices. (*L*) Min-to-max amplitude of the treatment interval normalized to the baseline for vehicle or SNAP-treated SCN comparing effects on PER2::LUC, [GABA]_e_ and [Ca^2+^]_i_. (*M*) Change in rhythmicity index (ΔRI) between baseline and treatment intervals of vehicle or SNAP-treated SCN for PER2::LUC, [GABA]_e_, and neuronal [Ca^2+^]_i_. (*N*) Ratio of [GABA]_e_ predicted/actual peak or trough amplitudes during vehicle or SNAP treatment. For *D*–*F*, N ≥ 4; *H*–*J*, N ≥ 3; *L*–*N*, N ≥ 7. In all plots, points represent SCN and bars represent mean ± SEM, except *B* where points represent genes.

To reconcile the apparently paradoxical observation that neuronal activity (which should drive GABA release) occurs when [GABA]_e_ is lowest and [GABA]_e_ is highest at night when SCN neurons are inactive, we investigated mechanisms that could control GABA flux. First, we explored published single-cell RNA-sequencing (scRNA-seq) data from SCN explants harvested during circadian day (CT7.5) or night (CT15.5) (13, 30) to determine the expression of genes encoding the four canonical GATs: GAT1 (*Slc6a1*, mGAT1), GAT2 (*Slc6a13*, mGAT3), GAT3 (*Slc6a11*, mGAT4), and BGT1 (*Slc6a12*, mGAT2). Data were clustered irrespectively of time of day into three principal cell groups: neurons (defined by *Slc32a1*, *Tubb3*, and *Celf4* expression), astrocytes (defined by *Aldh1l1*, *Gfap*, and *Aqp4* expression), and other (defined by exclusion of these markers). Cell clusters were then divided by time-of-day, and relative expression levels of the four *Gat* genes were evaluated. Genes encoding β-Tubulin 3 (*Tubb3*) and ALDH1L1 (*Aldh1l1*) were used as internal controls to confirm specific segregation of neuronal and astrocyte populations (Fig. 3*A*). This revealed *Gat1* and *Gat3* as the principal GATs within ex vivo SCN (with little to no *Gat2* or *Bgt1* expression), and expression of GAT1 and GAT3 across the SCN was confirmed by immunostaining (*SI Appendix*, Fig. S4*A*). scRNAseq revealed that *Gat1* was expressed by SCN astrocytes and neurons, while *Gat3* was specifically enriched in SCN astrocytes (Fig. 3*A*) (18, 31, 32). Furthermore, testing for differential expression in astrocytes between the day and night revealed a significant temporal variation in the levels of *Gat1* and *Gat3*, with expression being higher during the circadian day (Fig. 3*B*). This was confirmed by qPCR (*SI Appendix*, Fig. S4*B*). The fact that the transcriptomic data predict that GATs are present at a higher abundance during the circadian day when [GABA]_e_ is low and at a lower abundance when [GABA]_e_ is high highlights them as candidates to generate the observed dynamics in [GABA]_e_.

To determine the contribution of these transporters to the SCN TTFL oscillation, following an initial baseline recording we treated PER2::LUC explants with the GAT1-specific inhibitor CI966-HCl or the GAT3-specific inhibitor (S)-SNAP 5114 (Fig. 3*C*). Increasing dose of the GAT1 inhibitor CI966-HCl caused a small increase in period (Fig. 3*D*, one-way ANOVA F(6, 31) = 2.71, *P* = 0.03). This was accompanied by decreased precision (Fig. 3*E*, one-way ANOVA F(6, 31) = 14.01, *P* < 0.0001) and suppression of the amplitude (Fig. 3*F*, one-way ANOVA F(6, 38) = 26.95, *P* < 0.0001) with no lasting effect on the ongoing oscillation, which returned immediately following washout (*SI Appendix*, Fig. S5*A*). These changes occurred at relatively high concentrations, between 33 and 100 µM, however, which are >100-times the IC_50_ for CI966-HCl at GAT1 (IC_50_ 0.1 µM, ref. 33). Furthermore, at 100 µM, CI966-HCl could also inhibit GAT3 transporters (IC_50_ for GAT3 300 µM, ref. 33). We therefore interpreted this suppression as a consequence of (at least partial) inhibition of GAT3 alongside GAT1.

To clarify this, we recorded PER2::LUC rhythms from explants treated with a range of doses of the GAT3-specific inhibitor (S)-SNAP 5114 (Fig. 3*G*). Again, as dose increased, period lengthened (Fig. 3*H*, one-way ANOVA F(7, 36) = 9.40, *P* < 0.0001), precision decreased (Fig. 3*I*, one-way ANOVA F(7, 36) = 14.01, *P* < 0.0001), and amplitude was suppressed (Fig. 3*J*, one-way ANOVA F(7, 36) = 56.66, *P* < 0.0001). These effects were reversible, but in contrast to the immediate restoration following removal of CI966-HCl, the TTFL took several cycles to return to its initial state: amplitude progressively expanded, cycle-to-cycle, post-washout (*SI Appendix*, Fig. S5 *B* and *C*). Importantly, the concentrations of (S)-SNAP 5114 that maximally produced these sustained effects, between 33 and 50 µM, are within 6 to 10 times the IC_50_ for GAT3 transporters (IC_50_ 5 µM, ref. 34), thereby confirming the predominant role of GAT3 in sustaining TTFL function in the SCN.

To assess how treatment with (S)-SNAP 5114 alters the dynamics of [GABA]_e_, we recorded bioluminescent and fluorescent emissions from PER2::LUC SCN explants expressing *Syn.*iGABASnFr and *Syn.*jRCaMP1a, before and during treatment with either vehicle or 50 µM (S)-SNAP 5114 (Movies S2 and S3 and Fig. 3*K*). Vehicle had no effect, but GAT3 inhibition rapidly elevated [GABA]_e_ and suppressed neuronal [Ca^2+^]_i_ rhythms and PER2::LUC (Fig. 3 *K* and *L*). When quantified as the absolute peak-to-trough excursion, this suppression was significant relative to vehicle treatment across all reporters (Fig. 3*L*) (repeated-measures two-way ANOVA: treatment-effect F(1, 6) = 61.17, *P* = 0.0002; reporter-effect F(2, 12) = 2.40, *P* = 0.13; interaction F(2, 9) = 3.19, *P* = 0.09). Nevertheless, while rhythmicity was reduced across all reporters relative to vehicle (Fig. 3*M*) (repeated-measures two-way ANOVA treatment-effect F(1, 6) = 40.87, *P* = 0.0007), the effect was not equal across reporters (reporter-effect F(2, 12) = 5.69, *P* = 0.018; interaction F(2, 9) = 32.11, *P* < 0.0001). Post hoc multiple comparisons revealed that reduction by (S)-SNAP 5114 was most severe in the [GABA]_e_ oscillation (Sidak’s multiple comparisons test, DMSO vs. (S)-SNAP 5114 ΔRI: PER2::LUC 0.01 ± 0.03 vs. −0.16 ± 0.03 au, *P* = 0.007; [GABA]_e_, 0.01 ± 0.06 vs. −0.38 ± 0.04 au, *P* < 0.0001; [Ca^2+^]_i_ 0.06 ± 0.03 vs. −0.17 ± 0.03 au, *P* = 0.0003) (Fig. 3*M*). Chronic GAT3 inhibition therefore has potent effects on the ongoing TTFL oscillation through disruption of neuronal activity (as evidenced by [Ca^2+^]_i_ report), presumably through sustained elevation and severe disruption of the [GABA]_e_ rhythm.

GAT3 inhibition raised [GABA]_e_, reduced network rhythmicity, and eliminated [GABA]_e_ rhythmicity in SCN explants. The scRNAseq data suggested that the [GABA]_e_ rhythm is generated by a daytime GAT-mediated uptake of GABA from the extracellular space, meaning that GAT activity should account for the trough but not peak of [GABA]_e_. To test this, we extrapolated the trough and peak levels from baseline recordings forward into treatment intervals (*SI Appendix*, Fig. S6), enabling us to quantify the relative change in the peak and trough of the [GABA]_e_ rhythm whilst under treatment (Fig. 3*N*). This revealed a significant change in these metrics under treatment with (S)-SNAP 5114, but not vehicle (repeated-measures two-way ANOVA: treatment-effect F(1, 6) = 7.52, *P* = 0.03) which was not equal between the peak and the trough of the rhythm (repeated-measures two-way ANOVA: peak/trough-effect F(1, 6) = 10.13, *P* = 0.019; interaction, F(1, 4) = 9.02, *P* = 0.040). The flattening of the [GABA]_e_ rhythm was caused by elevation of the circadian trough, but not the peak of the oscillation (Sidak’s multiple comparisons test vehicle vs. (S)-SNAP 5114 actual/predicted ratio: peak 1.01 ± 0.01 vs. 1.10 ± 0.04 au, *P* = 0.25; trough 1.01 ± 0.02 vs. 1.22 ± 0.06 au, *P* = 0.01) (Fig. 3*N*). Thus, under free-running conditions, GAT3 in the SCN is responsible for taking up GABA released during the day, presumably to facilitate neuronal firing, which in turn is integral for high-amplitude, precise oscillation across the circuit.

### Daytime Uptake of GABA Permits Neuropeptidergic Signaling.

**Fig. 4.**
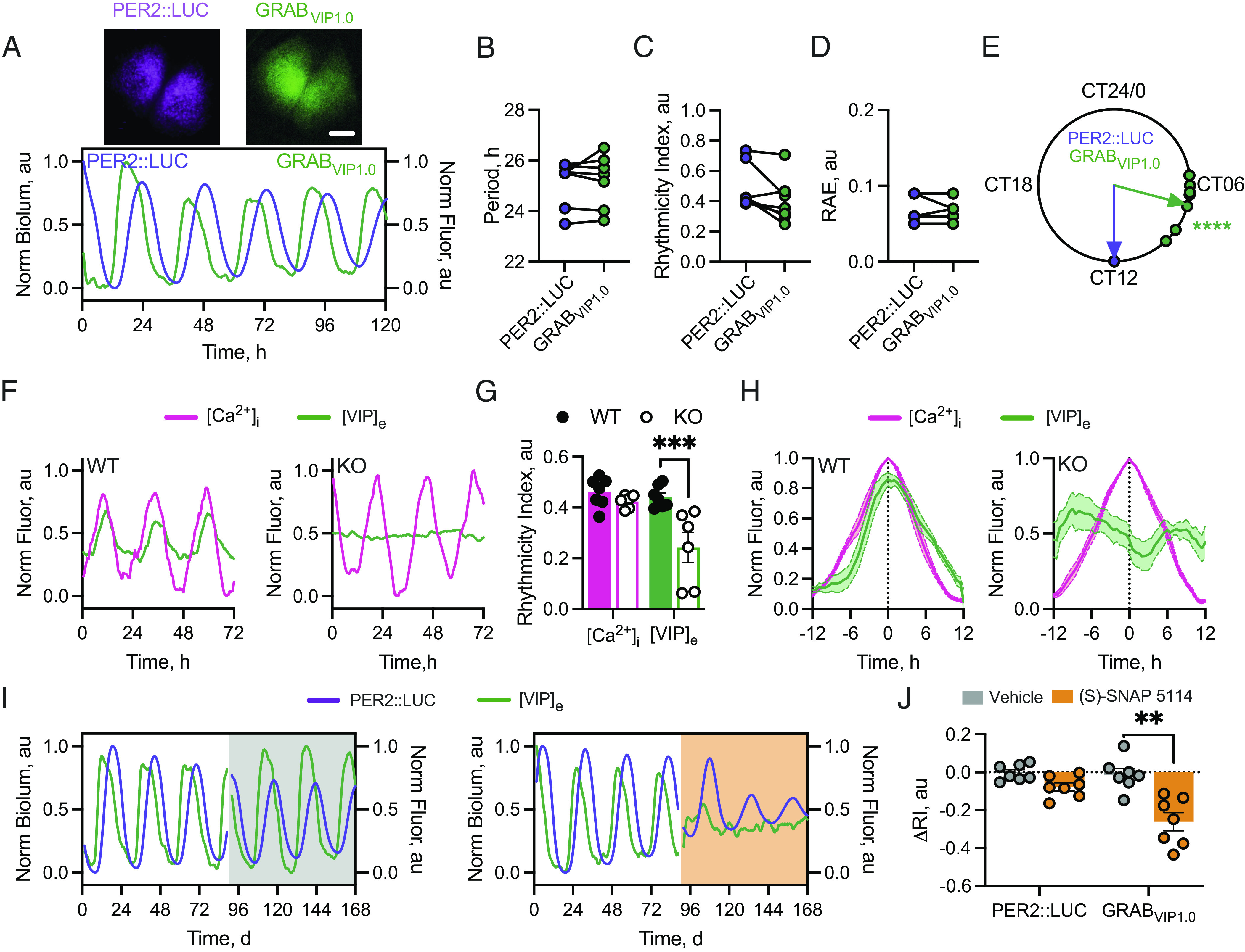
[VIP]_e_ in the SCN is rhythmic and is disrupted by GAT3 inhibition. (*A*, *Upper*) Average Z-projections of PER2::LUC (*Left*) and GRAB_VIP1.0_ (*Right*) in an ex vivo SCN slice. (*A*, *Lower*) Example normalized PER2::LUC and *Syn.*GRAB_VIP1.0_ fluorescence ([VIP]_e_). (*B*–*D*) Histograms showing aggregate statistics for PER2::LUC and [VIP]_e_ rhythms for period (*B*), rhythmicity index (*C*), and RAE (*D*). (*E*) Circular Rayleigh plot showing the relative phasing of the peak in [VIP]_e_ aligned on a slice-by-slice basis to peak PER2::LUC. (*F*) Example normalized fluorescence traces showing [VIP]_e_ alongside neuronal [Ca^2+^]_i_ from VIP-WT (*Left*) or VIP-KO (*Right*) SCN. (*G*) Histogram showing rhythmicity index of [VIP]_e_ and neuronal [Ca^2+^]_i_ in VIP-WT (filled circles/bars) or VIP-KO (open circles/bars) SCN. (*H*) Average normalized [VIP]_e_/[Ca^2+^]_i_ single-cycles aligned by [Ca^2+^]_i_ for VIP-WT (*Left*) and VIP-KO (*Right*) SCN. (*I*) Example PER2::LUC and [VIP]_e_ for vehicle (*Left*, gray) or 50 µM (S)-SNAP 5114 (SNAP) (*Right*, orange) treated SCN. (*J*) Change in rhythmicity index (ΔRI) between baseline and treatment intervals for PER2::LUC and [VIP]_e_ rhythms for vehicle and SNAP. For *B*–*E*, N = 7; *G*–*H*, N = 6 (WT and KO); *J*, N = 7. In all plots, points represent SCN (paired recordings are joined where relevant), bars, and lines/shading are mean ± SEM. (Scale bar, 250 µm.)

Our model predicts that the daily [GABA]_e_ rhythm, driven principally by daytime up-regulated GAT3 activity, facilitates increased neuronal excitability in the presence of enhanced neuronal GABA release. In the SCN, neuropeptide release is the critical functional consequence of neuronal excitability in sustaining circuit-level oscillations (35, 36). To interrogate the relationship between [GABA]_e_ and neuropeptide release, we recorded extracellular VIP levels ([VIP]_e_) via AAV-dependent expression of the fluorescent VIP sensor, GRAB_VIP1.0_ (*Syn*.GRAB_VIP1.0_). In this reporter, sensitivity to VIP is conferred by a catalytically dead VPAC2 receptor coupled to circularly permuted EGFP (37). We recorded *Syn.*GRAB_VIP1.0_ in SCN explants, phase-registering the signal against PER2::LUC oscillations (Fig. 4*A*). This revealed pronounced [VIP]_e_ rhythms with properties comparable to the PER2::LUC rhythm (Fig. 4 *B*–*D*, PER2::LUC vs. [VIP]_e_: period: 25.11 ± 0.35 h vs. 25.21 ± 0.39 h, paired two-tailed *t* test t(6) = 0.64, *P* = 0.55; rhythmicity index: 0.49 ± 0.06 vs. 0.41 ± 0.06, paired two-tailed *t* test t(6) = 2.42, *P* = 0.052; precision: 0.07 ± 0.01 au vs. 0.07 ± 0.01, paired two-tailed *t* test t(6) = 0.42, *P* = 0.69). Importantly, the rhythm in [VIP]_e_ peaked in mid-circadian day, CT6.97 ± 0.50 h (Fig. 4*E*, Rayleigh test R = 0.95, *P* < 0.0001) consistent with previous reports (36).

To confirm the specificity of the reporter, VIP-WT and VIP-null SCN were cotransduced with *Syn.*GRAB_VIP1.0_ and *Syn.*jRCaMP1a to enable phasing of [VIP]_e_ against neuronal [Ca^2+^]_i_. WT SCN displayed robust rhythms in [Ca^2+^]_i_ (Fig. 4*F*), whereas VIP-null SCN displayed damping [Ca^2+^]_i_ rhythms. Furthermore, VIP-null SCN displayed no observable [VIP]_e_ rhythm, with levels remaining at the limit of detection, thereby confirming reporter specificity (Fig. 4*F*). The absence of [VIP]_e_ rhythmicity in VIP-null SCN was reflected in the rhythmicity index (Fig. 4*G*) (repeated-measures two-way ANOVA: Genotype-effect F(1, 10) = 13.81, *P* = 0.004), where there was a reduced rhythmicity specifically in the [VIP]_e_ report (repeated-measures two-way ANOVA: reporter-effect F(1, 10) = 12.52, *P* = 0.005; interaction F(1, 10) = 6.70, *P* = 0.03). Consistent with these observations, when [VIP]_e_ was phase-aligned with [Ca^2+^]_i_ and plotted across a 24 h interval, VIP-null SCN again lacked a coherent [VIP]_e_ rhythm (Fig. 4*H*), whereas in WT SCN, the [VIP]_e_ peak was directly in phase with neuronal [Ca^2+^]_i_. This is consistent with the phase of peak membrane potential and [Ca^2+^]_i_ in VIP cells (~CT7) (8) and indicative of daytime neuronal activity driving VIP release. Importantly, this occurs when [GABA]_e_ is at its nadir, consistent with the view that low [GABA]_e_ is permissive for daytime neuronal activity and the dependent neuropeptide release.

To test this, SCN slices expressing GRAB_VIP1.0_ were treated with (S)-SNAP 5114 to examine the effects of loss of GAT3 function on VIP release in the SCN. Following baseline recording, slices treated with vehicle continued to show rhythmic GRAB_VIP1.0_ fluorescence (Fig. 4*I*). In contrast, in slices treated with (S)-SNAP 5114, the GRAB_VIP1.0_ rhythms were immediately compromised resulting in arrhythmicity (Fig. 4*I*), alongside a progressive damping of the PER2::LUC TTFL report. This was reflected in an immediate and severe reduction in the rhythmicity index in the [VIP]_e_ report (repeated-measures two-way ANOVA: reporter-effect F(1, 6) = 10.27, *P* = 0.0185) between baseline and treatment intervals (repeated-measures two-way ANOVA: treatment-effect F(1, 6) = 16.27, *P* = 0.0069; interaction F(1, 6) = 5.62, *P* = 0.0555) (Fig. 4*J*). GAT3 function is therefore necessary to sustain circadian daytime release of the neuropeptide VIP in the SCN.

### Astrocytes Initiate Network Rhythmicity through Acute Control of [GABA]_e_.

**Fig. 5.**
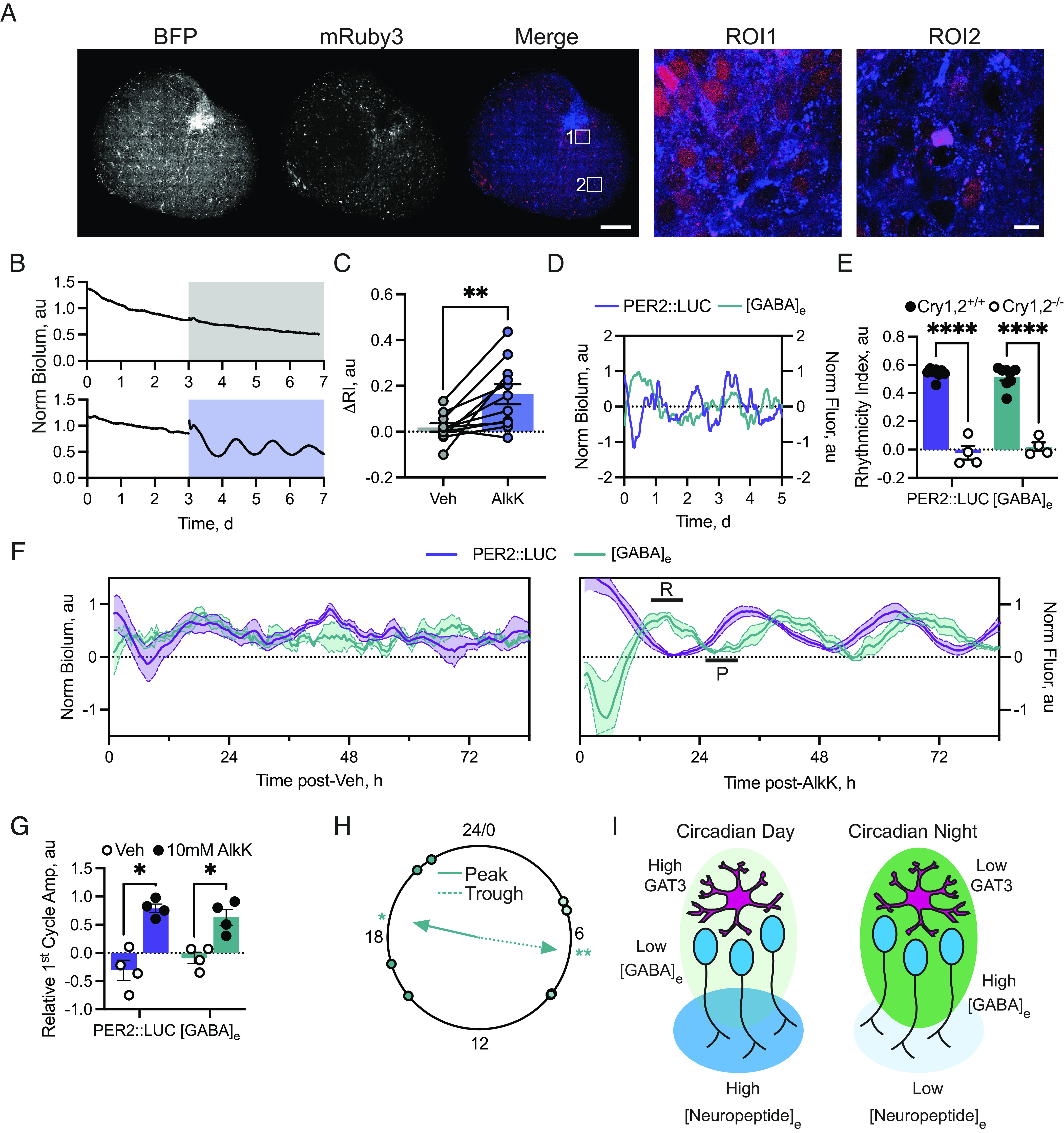
The astrocytic clock acutely controls [GABA]_e_ to transfer circadian information to the SCN network. (*A*) Confocal images of an SCN slice expressing *GFAP*.BFP2-P2A-mMPylS/PylT (*Left*, BFP) and CRY1_TAG_::mRuby3 (*Middle*, mRuby3) following 10 mM AlkK treatment alongside a false-coloured merged image [*Right*, Merge, BFP (blue) and mRuby3 (red)]. Two zoomed-in ROIs are shown to the right. (*B*) PER2::LUC traces from vehicle (*Upper*, gray) or 10 mM AlkK-treated (*Lower*, purple) CRY1,2-null SCN. (*C*) Change in rhythmicity index (ΔRI) between baseline and treatment intervals vehicle- or 10 mM AlkK-treated SCN. (*D*) Normalized PER2::LUC and [GABA]_e_ emissions from a CRY1,2-null SCN. (*E*) Histogram showing rhythmicity index measures for PER2::LUC and [GABA]_e_ from wild-type (filled circles, CRY1,2^+/+^, data from Fig. 1*C*) and CRY1,2-null (open circles, CRY1,2^−/−^) SCN. (*F*) PER2::LUC and [GABA]_e_ traces showing their dynamics during vehicle (*Left*) or AlkK treatment (*Right*). In the right-hand plot, phases where GABA is repressive or permissive are indicated by bars labeled R or P, respectively. (*G*) Histogram showing relative amplitude of the first cycle following treatment for PER2::LUC and [GABA]_e_ with vehicle (open circles) or 10 mM AlkK (filled circles). (*H*) Rayleigh plot showing timing of the first cycle trough (dotted line) and peak (solid line). (*I*) Schematic describing how the astrocytic clock controls network function via [GABA]_e_ flux. During circadian day (*Left*), astrocytic GAT3 expression is high, resulting in lower [GABA]_e_ (light green shading). This leads to increased neuronal activity and neuropeptide release (blue shading). During circadian night (*Right*), astrocytic GAT3 expression is low, resulting in high [GABA]_e_ (green shading) leading to reduced neuronal activity and neuropeptide release (light blue shading). In all plots, points represent SCN with paired measures joined where relevant. Bars and lines/shading represent mean ± SEM. For *C* N = 11, *E* N = 8 wild-type and 4 CRY1,2-null, *G* and *H* N = 4. (Scale bar, 200 µm/10 µm.)

Due to the fact that the GAT3 transporter is integral to neuropeptide release from SCN neurons to ensure correct network function and that it appears to be an astrocyte-enriched transporter and circadian-regulated, we next explored whether the circadian clock of astrocytes could, autonomously, control [GABA]_e_. We have previously used CRY1-complementation targeted to astrocytes to control circadian network function by initiating oscillations in arrhythmic CRY1,2-null SCN (38, 39). Using this approach, however, initiation takes >7 d (39). To increase the temporal precision with which the astrocytic TTFL could be controlled and its acute effects observed, we utilized translational switching (ts) of CRY1 protein (40). To validate this approach in astrocytes, CRY1,2-null SCN explants with the PER2::LUC reporter were transduced with tsCRY1 (*pCry1.*CRY1_(TAG)_::mRuby) (41) alongside the orthogonal tRNA-synthetase machinery targeted to astrocytes via *GFAP* promoter (*GFAP*.BFP2-P2A-mMPylS/PylT) (Fig. 5*A*). Following baseline recording, slices were treated with either vehicle or 10 mM noncanonical amino acid alkyne lysine (AlkK), a dose previously shown to initiate CRY1-translation in SCN explants (40–42). Vehicle-treated slices remained arrhythmic. In contrast, TTFL oscillation emerged in 10mM AlkK-treated slices (Fig. 5*B*) with a period (26.95 ± 0.27 h) appropriate for CRY1-driven oscillations. It was initiated within 2 d, faster than when Cre-recombinase is used to express CRY1 in astrocytes (38, 39). In all slices tested, 10 mM AlkK initiated SCN-wide rhythms as evidenced by the change in rhythmicity index between baseline and treatment intervals (Fig. 5*C*, ΔRI: vehicle 0.02 ± 0.02 vs. 10 mM AlkK 0.16 ± 0.04, paired two-tailed *t* test t(10) = 3.69, *P* = 0.0042). Furthermore, the effect was reversible on withdrawal of AlkK (*SI Appendix*, Fig. S7).

This approach therefore presented a unique opportunity to observe acute [GABA]_e_ changes as network oscillations were initiated by the cell-autonomous astrocytic TTFL. CRY1,2-null slices were supertransduced with *Syn.*iGABASnFR to monitor [GABA]_e_ as network rhythms initiated. In these CRY1,2-null SCN slices, similar to the TTFL rhythm, [GABA]_e_ was arrhythmic (Fig. 5 *D* and *E*) (repeated-measures two-way ANOVA: reporter-effect F(1, 10) = 0.11, *P* = 0.75) with a greatly reduced rhythmicity index in CRY1,2-null vs. wild-type slices (Figs. 1*C* and 5*E*, repeated-measures two-way ANOVA: Genotype-effect F(1, 10) = 300, *P* < 0.0001; interaction: F(1, 10) = 1.61, *P* = 0.23). Arrhythmicity was maintained following treatment with vehicle (Movie S4 and Fig. 5*F*). In contrast, under 10 mM AlkK treatment, not only were bioluminescent rhythms initiated but [GABA]_e_ also started to fluctuate with an initial rapid decrease followed by a rapid increase into a sustained peak (Movie S5 and Fig. 5*F*, marked R). This sustained elevation of [GABA]_e_ aligned with the nadir of the bioluminescent signal, marking the first circadian night and repressive phase of the TTFL clock (Fig. 5*F*). [GABA]_e_ then fell into a sharp trough (Fig. 5*F*, marked P) in the first circadian day, marked by rising PER2::LUC bioluminescence. Subsequently, [GABA]_e_ rose again to another sustained peak in the second circadian night (>CT12). These dynamics, therefore, align low [GABA]_e_ with the positive arm of the neuronal TTFL (38) and high [GABA]_e_ with the repressive arm. This indicates that, as network timekeeping is initiated, acute changes in [GABA]_e_ driven by the astrocytic TTFL generate network oscillation. They first facilitate repression via neuronal inhibition under high [GABA]_e_, which is then followed by coordinated facilitation of neuronal activity under low [GABA]_e_ via the permissive uptake of GABA from the extracellular space.

To assess the initiation of TTFL and [GABA]_e_ dynamics quantitively, we measured the coherence of the initiated oscillations by calculating their relative amplitudes, defined as the difference between the peaks and troughs predicted from the dynamics of the PER2::LUC bioluminescence recorded in photomultiplier tubes (PMTs) (Fig. 5*B*) and the circadian dynamics of [GABA]_e_ in wild-type slices (Fig. 1). We saw that in the high-temporal resolution PMT traces, PER2::LUC reached its first initiated peak 34.5 h (34.6 ± 0.6 h, Fig. 5*B*) after AlkK treatment. Using this, we identified when the troughs in the PER2::LUC and [GABA]_e_ oscillation that preceded this peak would have fallen (21 h and 26 h posttreatment, respectively) and where the ensuing peak in [GABA]_e_ would have fallen (39.5 h posttreatment) in the normalized data (Fig. 5*F*). Using these measures, we observed a low relative amplitude of the vehicle-treated oscillation in PER2::LUC and [GABA]_e_, which increased in both when treated with AlkK (Fig. 5*G*) (repeated-measures two-way ANOVA: reporter-effect F(1, 3) = 0.04, *P* = 0.85; treatment-effect F(1, 3) = 36.58, *P* = 0.009; interaction F(1, 3) = 0.18, *P* = 0.18). This indicates that the peak and trough of the [GABA]_e_ oscillation driven by the astrocytic clock were initiated immediately with dynamics corresponding directly with the [GABA]_e_ oscillation in a competent SCN (Figs. 1*A* and 5*H*) [Rayleigh test against specified mean direction (μ): peak (μ = CT19.7) *P* = 0.017, R = 0.73; trough (µ = CT8.2) *P* = 0.009, R = 0.80] and importantly occur acutely as network oscillations initiated. Thus, [GABA]_e_ is controlled by the cell-autonomous TTFL of astrocytes and encodes and transfers circadian information directly to the SCN neuronal network.

## Discussion

Free-running SCN explants display robust rhythms in [GABA]_e_, peaking at night in antiphase to neuronal activity (Fig. 1). This observation is counterintuitive because a peak in [GABA]_e_ would be expected in circadian day when SCN GABAergic neurons are active. Equally, GABA is inhibitory, and so, high daytime [GABA]_e_ would be expected to suppress neuronal activity and therefore its own release. The paradox could be resolved if GABA were excitatory, a condition observed during development (43), arising when intracellular chloride levels ([Cl^−^]_i_) are high. This is achieved via changes in the expression of K-Cl (KCC2, *Slc12a5*) and Na-K-2Cl (NKCC1, *Slc12a2*) cotransporters and leads to a reversal of GABA_A_-receptors on activation (44, 45). Indeed, a model whereby SCN neurons may switch GABAergic transmission between excitatory and inhibitory on a circadian basis has been proposed (46) and excitatory GABAergic drive reported in the SCN under certain conditions. There is, however, no consensus on its temporal or spatial patterning (for review see ref. 17). More directly, our data contradict this model, indicating that GABAergic signaling, mediated by GABA_A_-receptors, forms a suppressive rather than excitatory axis in the SCN (Fig. 2 and *SI Appendix*, Figs. S2 and S3). In our explants, the SCN neurons and astrocytes demonstrate mature phenotypes, and there is no evidence in our preparations for the activity of NKCC1 (*SI Appendix*, Fig. S8). GABA is therefore suppressive, and this suppression is presumably driven by extra-synaptic GABA_A_-receptors which are well suited to sense circadian changes in bulk [GABA]_e_ and drive long-term inhibition during the night due to their high affinity and decreased desensitization in the prolonged presence of GABA (17, 47, 48). A limitation of our approach, however, is that we have not assessed this mechanism directly via electrophysiological investigation. A wealth of electrophysiological data focusing on the specifics of cellular GABAergic signaling in the SCN are available (17, 49), but here our focus was specifically on the global dynamics of [GABA]_e_.

While we consider GABA to be suppressive within the SCN, via a potentially tonic mechanism, this could have effects beyond simple inhibition of the neurons. In thalamic neurons, a consequence of increased tonic GABAergic currents is to hyperpolarize neurons into the activation range for T-type calcium channels (Cav3.1, 3.2, and/or 3.3) (50), which serves to permit neuronal activity through rebound depolarization (51). Additionally, in hippocampal neurons, it has been shown that activation of tonic GABAergic currents decreases the membrane time-constant leading to a requirement for higher precision of timing of incoming excitation (52). In both these ways, enhanced tonic GABAergic currents alone or in tandem with T-type calcium channels can generate excitation and augment responses to incoming afferent signals to synchronize and promote neuronal activity. T-type channels are expressed within the SCN (53–55), although in isolated SCN, pharmacological inactivation of T-type calcium channels does not alter the circadian electrophysiological properties of SCN neurons (54). This indicates that activation of T-type calcium channels is not required at network steady state to generate or synchronize circadian cycles of neuronal firing. This does not preclude a role for interaction between tonic GABA and T-type calcium channels, as this could be a mechanism in vivo by which responses to incoming glutamatergic afferents from outside the SCN are augmented by T-type calcium channels during the night. Indeed, pharmacological inactivation of these calcium channels has been shown to block glutamate-induced phase delays during circadian night (53) when [GABA]_e_ is rising (Fig. 1). Our current model therefore predicts that the high level of GABA we report during the night generates tonic GABA currents that act at two levels: first, it supresses neuronal activity when the network is at steady state, and second, it potentially primes the network to respond to appropriately timed extra-SCN afferent signals through a mechanism that involves T-type calcium channels.

In our model, astrocytes, not neurons, control [GABA]_e_ (Fig. 5*I*). This predicts that GABA uptake during the day (when neurons are most actively releasing GABA) via the GAT3 transporter prevents GABA spill-over from synaptic sites and accounts for the sharp trough in the [GABA]_e_ oscillation (Fig. 1). This uptake is permissive to neuronal activity, which in turn supports electrically sensitive CREs in *Per* genes, thereby leading to robust high-amplitude TTFL oscillation across the SCN (Fig. 2) (3, 4). Conversely, elevated [GABA]_e_ sustains the nocturnal repressive phase of the TTFL.

How then, does the [GABA]_e_ rhythm sit in antiphase to the activity of its presumed source? Using our published single-cell transcriptomic dataset (13), we found a day–night difference in GAT expression, predominantly on astrocytes (Fig. 3 and *SI Appendix*, Fig. S3). SCN expression of GATs has been reported (18, 31, 32) and potential circadian variation shown in rat SCN (18) and cortical astrocytes (31). Further, this circadian difference in GAT transcripts has been reported in cortical and hippocampal preparations to be controlled by the circadian transcription factor REV-ERBα via inhibition of the clock-controlled output gene E4BP4 (56), driving transcript levels to peak during the day and reach their nadir during the night, consistent with our proposed model. Our functional tests of GAT1 and GAT3 inhibition revealed that their inactivation suppressed the PER2::LUC oscillation (Fig. 3) because of the consequent rising levels of GABA_A_-receptor activation (Fig. 2). Furthermore, GAT3 inhibition produced stronger effects on application and during washout (Fig. 3 and *SI Appendix*, Fig. S5), likely due to the fact that GAT3 has a higher affinity for GABA compared to GAT1 (57) allowing GAT3 to lower GABA levels more efficiently in the absence of GAT1 function. GAT3 is therefore the stronger candidate as the principal transporter for control of [GABA]_e_ and network function in the SCN via daytime GABA uptake. This critical daytime uptake of GABA was confirmed by direct imaging of the [GABA]_e_ oscillation under treatment with the GAT3-specific inhibitor (S)-SNAP 5114, where loss of rhythmicity in the GABA report is caused by an acute elevation of the trough without effects on peak level (Fig. 3). Daytime removal of GABA from the extracellular milieu is therefore necessary for neuronal activity, and activity-dependent neuropeptide release, which reinforces circuit-level rhythms (Fig. 4) accounting for the loss of PER2::LUC amplitude.

Consistent with our model, whereby a low level of [GABA]_e_ is permissive to timekeeping, sustained loss of GABAergic release from neurons by genetic targeting of the vesicular transporter VGAT does not markedly influence SCN molecular rhythmicity (26), even though it compromises transmission of behaviorally relevant cues at distal extra-SCN targets. Equally, TTFL function in the SCN is sustained during blockade of GABA_A_- and/or GABA_B_-receptors (Fig. 2 and refs. 9 and 25), although GABA_A_ blockade [(+)-bicuculline] elevates both the peak and trough of the oscillation (Fig. 2), indicating that GABA fine-tunes SCN electrical activity within a certain dynamic range that complements TTFL function (26, 27). The nocturnal rise in [GABA]_e_ is important because it curtails the permissive low-GABA state. If that low-GABA state is prevented by GAT3 blockade, not only is the neuronal activity rhythm (monitored by [Ca^2+^]_i_) damped but the consequent release of neuropeptides is obstructed.

VIP is a principal SCN neuropeptide, essential for circadian neuronal synchrony and high-precision oscillation (58–60), driving the cell-autonomous TTFL of cells expressing its receptor, VPAC2, by kinase-dependent signaling to CRE-elements in the *Per* genes (7, 61). A VIP-GABA counterbalance mechanism has been proposed to regulate the SCN TTFL (20, 31) and GAT-mediated GABA uptake suggested as a conduit for circadian astrocyte-to-neuron information transfer in coculture in vitro models (31). Indeed, pharmacological disruption of GABAergic signaling counteracts some of the deficits of VIP deficiency in isolated SCN (20), adding to the notion that opposing GABAergic and neuropeptidergic cues set the dynamic range for normal TTFL function. Using a VIP-specific reporter (37), we mapped peak VIP release to the middle of circadian day (CT6.97), consistent with previous recordings and the known activity cycle of VIP neurons (8, 36) and showed that VIP and GABA are ordinarily maintained in a distinct temporal segregation in the SCN, held in antiphase by GABA uptake by astrocytic transporters. Compromising this uptake rapidly damped rhythmic VIP release, revealing explicitly the interplay between [GABA]_e_ and [VIP]_e_ in circadian time and showing that control of [GABA]_e_ not only directs the cell-autonomous TTFL but also circuit-level SCN timekeeping.

Nighttime release of GABA is compatible with our previously proposed astrocyte-to-neuron signaling axis, whereby nocturnally released astrocytic glutamate depolarizes neurons expressing NR2C-containing NMDA receptors, increasing action potential-independent GABA release from these neurons (6). The effectiveness of this synaptic release would be augmented by nocturnal inactivation of GABA uptake, allowing for enhanced spill-over of GABA into extra-synaptic sites. A further intriguing aspect is that the metabolism of GABA and glutamate is intimately interlinked via the GABA/glutamate–glutamine shuttling between astrocytes and neurons (62), which may point to some metabolic coupling between the daytime active neurons and nighttime active astrocytes. Indeed, pharmacological disruption of astrocytic metabolism compromises the ongoing network oscillation (39). In the SCN, synthesis of glutamate is dependent on astrocytic metabolism, positioning them as the metabolic source (6). However, GABA metabolism is more complex, and it has been shown in other brain areas that both neurons [which express GAD65/67 (63, 64)] and astrocytes are capable of synthesizing GABA, potentially via alternative mechanisms (65). Release of glutamate into the SCN extracellular space has been tracked to nonvesicular release from the astrocytes (6, 38), but whether the source of the nighttime GABA is solely from the neurons or also arises from the astrocytes by similar mechanisms remains unknown. Nevertheless, in these complementary, coordinated ways, it is evident that astrocytes can suppress the SCN neuronal network during the night directly by manipulating the properties of their extracellular milieu.

Evidence of this reciprocal astrocyte-to-neuron signaling is provided by the reemergence of TTFL rhythms following the removal of GAT3 inhibition. On washout, the TTFL amplitude did not return immediately, but rather, it grew steadily, cycle-to-cycle (*SI Appendix*, Fig. S5 *B* and *C*). This progressive recovery following a switch from high [GABA]_e_ to low [GABA]_e_ can be explained by the mutual dependence of neuronal and astrocytic TTFLs. In the prolonged high [GABA]_e_ state, loss of neuronal rhythmicity likely also impairs astrocytic rhythms secondarily, and following transfer to the low [GABA]_e_ state, it requires several cycles of mutual reinforcement to establish full spectrum oscillations in both cell types. A comparable reestablishment of TTFL cycles occurs following washout of cycloheximide (suspending cell-autonomous TTFLs) or tetrodotoxin (suspending neuronal signaling) (10, 66, 67). In the case of GAT3 inhibition, these dynamics are not, however, driven purely by release of GABAergic inhibition to the neurons because washout of muscimol does not produce the same delayed increase in amplitude (*SI Appendix*, Fig. S2). The building of amplitude upon washout of GAT3 therefore represents a gradual reconfiguration of the mechanisms to control GABA uptake and release across neurons and astrocytes following chronic inactivation of an astrocyte-specific transporter. We therefore interpret this as the SCN network iteratively rebalancing itself as the neurons and astrocytes are recoupled on a cycle-to-cycle basis. Comparable washout dynamics are seen following disruption of astrocyte-to-neuron signaling when NR2C-receptors or glutamate uptake are inhibited and then restored (6), which could reflect mutual recoupling of signaling and/or metabolic processes.

Our model proposes that daily rhythms in GABA uptake, driven principally by astrocytes, control circadian dynamics of bulk GABA in the extracellular space. This uptake is dependent on a functional SCN clock, as [GABA]_e_ is arrhythmic in CRY1,2-null explants, consistent with previous observations of arrhythmic astrocytic TTFL and [Ca^2+^]_i_ and extracellular glutamate (38, 39, 68). In order to identify causal events in a repetitive, cyclical system such as the SCN, where changes on one cycle may be caused by events some cycles previously, it is necessary to have precise temporal control of the pertinent components. We therefore used tsCRY1-expression specifically in astrocytes to activate their cell-autonomous TTFL and thereby test our model by following the sequence of events (Fig. 5) as circadian rhythmicity was initiated, de novo, in arrhythmic CRY1,2-null SCN (40–42). This revealed precise patterning of coherent [GABA]_e_ dynamics occurring over the first 48h, with changes in [GABA]_e_ simultaneous with those in the TTFL. Furthermore, from their initiation stage, these rhythms matched the relative phasing in wild-type SCN. Thus, in the absence of a functional clock in the rest of the SCN, astrocytes can impose their cell-autonomous circadian state onto the SCN network by initiating circadian cycles of [GABA]_e_ which are necessary to entrain the “clockless” neurons. We have, therefore, revealed a different level of astrocyte-to-neuron communication that controls SCN network dynamics. Having established this mode of communication, it is now imperative to explore whether further signaling axes support this and to determine the mechanisms by which neurons reciprocate this signaling to astrocytes. A particularly striking observation from our results is that despite neurons being the (presumed) source of GABA in the SCN network, circadian control of this signaling is devolved to the astrocytes. Is this, therefore, a more general mechanism whereby astrocytes regulate other GABAergic circuits in the brain across different time-scales?

## Materials and Methods

Detailed description of materials and methods is provided in *SI Appendix* along with a summary of analysis techniques and R scripts to repeat scRNA-seq analysis of publicly available datasets.

Animals were used in accordance with the UK Animals (Scientific Procedures) Act of 1986, with local ethical approval (MRC LMB AWERB). SCN explants were made from postnatal day 10 (P10) to P12 of either sex and cultured as an organotypic explant via the interface method. Slices cut at 300 µm were rested for at least a week before further experimentation or AAV transduction. AAV transductions were carried out by applying AAV particles in PBS as a 1 µL droplet to the surface of the SCN explant. In the case of serial transductions, these were made 2 to 3 d apart and explants were left for at least 1 wk following the final transduction before the medium was changed. Full details of the animal genotypes and AAVs used are detailed in *SI Appendix*.

PER2::LUC bioluminescence was recorded in PMTs or a LumiCycle (Actimetrics), while multiplexed bioluminescence and fluorescence imaging was carried out on LV200 Bioluminescence Imaging Systems (Olympus). Where slices were manipulated pharmacologically, compounds were added directly to the medium. For confocal imaging, SCN explants were fixed in 4% PFA before subsequent processing for immunohistochemistry and mounting. Full details of the recording procedures and parameters for live bioluminescent and fluorescent recordings and confocal fixed imaging are included in *SI Appendix*.

For qPCR analysis, RNA was extracted from explants (phase-mapped via their PER2::LUC oscillation) using a Direct-Zol RNA microprep kit column-based kit (Zymo Research) and reverse transcribed to cDNA using the QuantiTect Reverse Transcription Kit (Qiagen). qPCR was carried out using the QuantiNova SYBR Green PCR Kit (Qiagen), and all samples and the standard curve were run in triplicate alongside no template controls on a Techne PrimePro 48 real-time PCR machine (Techne). All primers, cycling parameters, and associated information are included in *SI Appendix*.

## Supplementary Material

Appendix 01 (PDF)

Movie S1. **Example recording of PER2::LUC and *Syn*.iGABASnFR (related to Fig. 1)**. Example video of an *ex vivo* SCN explant showing bioluminescent PER2::LUC emissions (left) alongside *Syn*.iGABASnFR fluorescence (right).

Movie S2. **Vehicle treated recpording of PER2::LUC, *Syn*.jRCaMP1a and *Syn*.iGABASnFR (related to Fig. 3)**. Example video of an *ex vivo* SCN explant showing bioluminescent PER2::LUC emissions (left) alongside *Syn*.jRCaMP1a (middle) and *Syn*.iGABASnFR (right) fluorescence. An initial baseline interval is shown, followed by vehicle treatment as denoted in the upper left corner (+ Vehicle).

Movie S3. **(S)-SNAP 5114 treated recording of PER2::LUC, Syn.jRCaMP1a and Syn.iGABASnFR (related to Fig. 3)**. Example video of an *ex vivo* SCN explant showing bioluminescent PER2::LUC emissions (left) alongside *Syn*.jRCaMP1a (middle) and *Syn*.iGABASnFR (right) fluorescence. An initial baseline interval is shown, followed by 50μM (S)-SNAP 5114 treatment as denoted in the upper left corner (+ 50μM (S)-SNAP5114).

Movie S4. **Vehicle treated CRY-null recording of PER2::LUC and *Syn*.iGABASnFR (related to Fig. 5)**. Example video of a vehicle treated *ex vivo* CRY-null SCN explant expressing translationally switchable *pCry1*.CRY1::mRuby3 and astrocytically-targeted tRNA synthetase showing bioluminescent PER2::LUC emissions (left) alongside *Syn*.iGABASnFR (right) fluorescence. Vehicle was added to the medium immediately before recording started.

Movie S5. **AlkK treated CRY-null recording of PER2::LUC and *Syn*.iGABASnFR (related to Fig. 5)**. Example video of an AlkK treated *ex vivo* CRY-null SCN explant expressing translationally switchable *pCry1*.CRY1::mRuby3 and astrocytically-targeted tRNA synthetase showing bioluminescent PER2::LUC emissions (left) alongside *Syn*.iGABASnFR (right) fluorescence. 10mM AlkK was added to the medium immediately before recording started.

## Data Availability

All study data are included in the article and/or supporting information. Previously published data were used for this work [NCBI Gene Expression Omnibus with the accession number: GSE167927 (https://www.ncbi.nlm.nih.gov/geo/query/acc.cgi?acc=GSE167927)] (30).
