# Supplementary material for: Astrocytic control of extracellular GABA drives circadian timekeeping in the suprachiasmatic nucleus
Source: Proc Natl Acad Sci U S A. 2023 May 15;120(21):e2301330120. doi: 10.1073/pnas.2301330120 (PMC10214171; doi:10.1073/pnas.2301330120)
Supplement: Supplementary file 1 — Appendix 01 (PDF) [file pnas.2301330120.sapp.pdf]

PNAS

Supporting Information for

Astrocytic control of extra-cellular GABA drives circadian time-keeping in the suprachiasmatic nucleus.

Andrew P. Patton*, Emma L. Morris, David McManus, Huan Wang, Yulong Li, Jason W. Chin and Michael H. Hastings*

*To whom correspondence may be addressed. Email: mha@mrc-lmb.cam.ac.uk and apatton@mrc-lmb.cam.ac.uk

This PDF file includes:

Supporting text
Figures S1 to S8
Supplementary Movie Legends
SI References

Supporting Information: Materials and Methods

Experimental model and subject details

Animals

All experiments were performed in accordance with the UK animals (Scientific Procedures) Act of 1986, with local ethical approval (MRC LMB AWERB). PER2::Luciferase (PER2::LUC) mice were kindly supplied by J.S. Takahashi University of Texas Southwestern Medical Center, Dallas, TX; (1). VIP-null mice were a gift from C.S. Colwell; (2). CRY1,2-null mice were derived from founders supplied by G. van der Horst (Erasmus University Medical Center, Rotterdam, The Netherlands; (3)). CRY1,2-null mice were crossed to the PER2::LUC line in-house and all mice were maintained on a C57BL6/J background.

Organotypic slice preparation and AAV transduction

Postnatal day 10 (P10) to P12 mice of either sex were killed according to local and UK Home Office approved rules. The brains were removed and transferred to ice-cold GBSS supplemented with 5mg/ml glucose, 3mM $MgCl_2$, 0.05mM DL-AP5 and 100nM (+)-MK-801. Brains were trimmed into a block containing the hypothalamus before 300μm thick coronal slices were made on a tissue chopper (McIlwain, UK). The SCN was then dissected free from the slice and cultured as an organotypic explant via the interface method. Slices were rested in culture medium supplemented with 3mM $MgCl_2$, 0.05mM DL-AP5 and 100nM (+)-MK801 before being transferred to medium for 1 week before use (4).

Method details

AAVs and molecular biology

The following AAVs were obtained as viral preps directly from Addgene. pAAV.*hSynap*.iGABASnFR (*Syn*.iGABASnFR, AAV1) was a gift from Loren Looger (Addgene viral prep # 112159-AAV1; http://n2t.net/addgene:112159; RRID:Addgene_112159) (5). pAAV.*Syn*.NES.jRCaMP1a.WPRE.SV40 (*Syn*.jRCaMP1a, AAV1) was a gift from Douglas Kim & GENIE Project (Addgene viral prep # 100848-AAV1; http://n2t.net/addgene:100848; RRID:Addgene_100848) (6). The following AAV was obtained as a plasmid from Addgene, and packaged as into AAV serotype 1 viral particles by VectorBuilder (https://www.vectorbuilder.com/). pAAV.*hSynap*.ArcLightD.WPRE.SV40 (*Syn*.ArcLight) was a gift from Vincent Pieribone (Addgene plasmid #100037; http://n2t.net/addgene:10037; RRID: Addgene_100037) (7).

hSyn.$GRAB_{VIP1.0}$.WPRE was a gift from Yulong Li (School of Life Sciences, Peking University, China) and was obtained as a plasmid before being packaged into AAV serotype 9 viral particles by VectorBuilder (8). tsCRY1::mRuby (*pCry1*.CRY1(TAG)::mRuby3) was produced in-house and packaged by VectorBuilder as AAV1 serotype AAV particles as detailed previously (9). *GFAP*.BFP2-P2A-MmPylS was made in-house by swapping the fluorescent tag in the *GFAP*.mCherry-P2A-MmPylS plasmid (10) for BFP2 before being packaged by VectorBuilder as AAV serotype 5 viral particles.

AAV transduction

In the case of SCN slices that received AAV transductions, following at least a week in culture, the medium was changed and slices were transduced with 1-2μl of AAV (>$1x10^{12}$ genome copies/ml in PBS) applied directly as a droplet to the top of the SCN. In the case of SCN receiving multiple serial transductions, 2-3 days were left between each round of transduction. A week following the final transduction, the medium was changed.

Real-time bioluminescent and fluorescent imaging

Aggregate bioluminescence was monitored in real-time in customised light-tight incubators fitted with individual photon-multiplier tubes (PMTs; catalog #H9319-11 photon counting head, Hamamatsu). Data were binned into 6-minute epochs for export and analysis. Slices were maintained in a HEPES-buffered medium containing DMEM, supplemented with Glutamax,

penicillin/streptomycin, FCS, B27 and 100µM luciferin as detailed previously (4). Dishes were sealed with glass coverslips.

For combined circadian fluorescent/bioluminescent imaging, SCN slices were sealed in glass-bottomed imaging dishes (P35G-0-10-C, Mattek) containing the same medium as in PMT recordings. Slices were imaged on an LV200 Bioluminescence Imaging System (Olympus). Bioluminescence was acquired for between 9.5 and 29.5 mins, while multiplexed fluorescence acquisition was set at 100ms. The acquisition intervals for combined circadian imaging were 30 mins.

Immunostaining and Confocal Microscopy
Membrane-attached SCN slices were excised via scalpel and fixed by submersion in 4% paraformaldehyde (PFA) diluted in phosphate buffer for 30 minutes at room temperature. Following this, slices were washed twice for 10 minutes each in 0.01M phosphate buffered saline (PBS) at room temperature. Washed slices were transferred to a blocking buffer composed of 0.01M PBS supplemented with 1% bovine serum albumin (BSA), 0.5% Triton X-100 and 5% Normal Goat Serum (Day 1 buffer) and blocked for 6 hours at room temperature before being transferred to Day 1 buffer supplemented with primary antibodies/antisera and incubated overnight at 4°C. Following incubation with the primary antibodies/antisera, slices were washed twice in Day 1 buffer diluted 1/3 in 0.01M PBS (Day 2 buffer) for 15 minutes each, at room temperature. Slices were then transferred to the Day 2 buffer supplemented with the secondary antibodies and incubated for 1 hour at room temperature. Following incubation with the secondary antibodies, slices were washed twice in Day 2 buffer and twice in 0.01M PBS for 15 minutes each at room temperature. Finally, slices were rinsed in ddH_2O and mounted on glass slides before being coverslipped with Vectamount HardSet antifade mounting medium (H-1400, Vector Laboratories). Where fluorophores permitted, the mounting medium used was supplemented with DAPI (H-1500, Vector Laboratories). All incubation and wash steps were carried out on a shaker.

The following primary antisera were used at the following dilutions: Rabbit anti-GAT1 (Synaptic Systems, 274 102) (1:500) and Guinea Pig anti-GAT3 (Synaptic Systems, 274 304) (1:500). The following secondary antibodies were used at the following dilutions: Goat anti-Rabbit Alexa 488 (Invitrogen, A11008) (1:1000) and Goat anti-Guinea Pig 568 (Invitrogen, A11075) (1:1000).

Mounted immunostained SCN were imaged on a Zeiss 880 Airyscan confocal microscope controlled by Zen software (Zen 2.3, Zeiss). Imaging was carried out with a 20x apochromatic objective and the whole of the SCN slice was captured in tiles at 3 different focal planes due to the uneven thickness of cultured SCN explants. Post-imaging, Z-projections were made of the maximum pixel intensity to create a composite image of the entire SCN explant.

In the case of imaging native fluorescent markers expressed via AAVs, SCN were fixed, washed and mounted before being imaged on a Zeiss 880 Airyscan confocal microscope with a 63x oil-immersion apochromatic objective to enable better resolution imaging of the fluorescent proteins. In all cases, slices were imaged as tiles to capture the whole of the SCN.

Pharmacological treatments
(R)-baclofen, (+)-bicuculline, bumetanide, CI 966 HCl, DMSO, muscimol, SCH 50911 and (S)-SNAP 5114 were obtained from Tocris, UK. (R)-baclofen and muscimol were solubilised in water to stock solutions of 20mM and 100mM respectively. (+)-bicuculline, bumetanide, CI 966 HCl, SCH 50911 and (S)-SNAP 5114 were solubilised in DMSO at a stock concentration of 100mM (with the exception of bumetanide which was at 10mM and CI 966 HCl which was at 50mM). The corresponding vehicle (water or DMSO) was used with the corresponding pharmacological agent and for dose-response curves, dilutions were made in the same vehicle as serial dilutions. Pharmacological agents were washed out by transferring the membrane and SCN to fresh media three times serially, waiting for 10 minutes at each step.

Alkyne lysine (AlkK, N6-2-propynyloxycarbonyl-l-lysine, synthesised in-house) was prepared freshly as a stock solution of 100mM dissolved directly in recording medium, before being adjusted to pH7.0 by addition of NaOH, as previously described (9, 11, 12). The AlkK solution was then sterile filtered before being added to slice medium at a 1:10 dilution to result in a final concentration of 10mM in the recording medium. For vehicle treatments, a 1:10 dilution of medium without AlkK was added to the recording medium. AlkK or vehicle were washed out of the slices by transferring the membrane and SCN to fresh media six times serially, waiting for 10 minutes at each step.

RNA extraction and cDNA generation

SCN slices were harvested during two specific phase windows: CT0-4 (circadian day) and CT12-16 (circadian night). Circadian phasing of the slices was made by recordings PER2::LUC emissions in a LumiCycle (Actimetrics), with the data collected in the LumiCycle software (version 6.106, Actimetrics) and exported using the LumiCycle Analysis software (version 3.101, Actimetrics). The circadian phase was assessed by calculating the difference in time between the previous peak in the oscillation and the time at which the slice was collected, normalised to the circadian period of the oscillation. Individual SCN slices were detached from the culture membranes by gentle manipulation with fire-blunted borosilicate glass while submersed in 0.01M PBS before being transferred via pipette to individual tubes.

RNA extraction from SCN slices was carried out using a column based Direct-zol RNA microprep kit (R2060, Zymo Research). Excess PBS was removed, and slices were disrupted and lysed in 100µl TRI Reagent (R2050, Zymo Research) by vortexing at full speed for 1 minute. The rest of the purification steps were carried out according to the manufacturers protocol, including on-column DNase I treatment. Following the final wash step, the column was spun in a clean tube for an additional 3 minutes to ensure complete removal of the wash buffer. RNA was eluted by the addition of 14µl DNase/RNase-free water which was left to stand for 3-5 minutes before centrifugation for 1 minute. RNA concentration and purity were checked on a nanodrop before reverse transcription.

RNA was reverse-transcribed using QuantiTect Reverse Transcription Kit (205310, Qiagen). First, 12µl of purified RNA was incubated with 2µl gDNA Wipeout Buffer at 42ºC for 2 minutes to ensure complete removal of any remaining genomic DNA as per manufacturer instructions. Following this step, the entire reaction mix was incubated with Quantiscript reverse transcriptase, Quantiscript RT Buffer and RT primer mix in a total reaction volume of 20µl at 42ºC for 30 minutes before being inactivated at 95ºC for 3 minutes. Samples were immediately placed on ice and were then diluted 1:2 with water to yield a final volume of 40µl.

qPCR

Quantative PCR (qPCR) was carried out using QuantiNova SYBR Green PCR Kit (208052, Qiagen), as per manufacturer instructions. Each individual PCR reaction contained: 1x SYBR Green PCR Master Mix, 1x QN ROX Reference Dye, 0.7µM Forward Primer and 0.7µM Reverse Primer. Primers either were designed in-house using the IDT PrimerQuest tool (https://eu.idtdna.com/pages/tools/primerquest) or were obtained from the GETPrime2.0 database (https://gecftools.epfl.ch/getprime) (13) and were synthesised by Millipore. Primer sequences were:

Gene	Primer Sequence	Source
Rn18s F	5'-CAGTAAGTGCGGGTCATAAG-3'	In house, designed using IDT Primer Quest.
Rn18s R	5'-CTAAACCATCCAATCGGTAGTAG-3'	
Slc6a1 F	5'-CCATGTAGCAAAGCGTATGT-3'	In house, designed using IDT Primer Quest.
Slc6a1 R	5'-CGGTCACAGTTATTCACCATAG-3'	
Slc6a11 F	5'-CATGCTGTGTATCCCACTC-3'	GETPrime2.0 Database.
Slc6a11 R	5'-GTCAACTTCTGTAATTTCTCGG-3'	

In order to generate a standard curve, 10µl of each SCN sample being assessed in a run were pooled together to make a 100% standard sample. Subsequently, 5 serial dilutions at 1:5 were

made to create 5 standards in total: 100%, 20%, 4%, 0.8% and 0.16%. Finally, each individual SCN cDNA sample was diluted 1:5 so that its concentration would fall within the range of the standard curve. 2.5µl of cDNA samples were added to each well (alongside no template controls) and all samples were run in triplicate. The plates were run on a Techne PrimePro 48 real-time PCR machine (Techne) using the Eco 48 software (v5.0, PCRMax) to acquire data. All samples were run with the following thermal profile: 2 mins at 95ºC followed by 40 cycles of 95ºC for 10s and 60ºC for 15s before a melt-curve was run between 95ºC and 55ºC.

Data was analysed in the EcoStudy software (v5.0, PCRMax) to determine the cycle threshold (CT) values for the standard curves and the individual samples. Primer efficiencies were determined from the standard curve, and ranged between 94% and 98%. Relative expression was calculated in excel using the Pfaffl method (14) and 18S ribosomal RNA (*Rn18s*) was used as the reference gene.

Quantification and statistical analysis

Analysis of real-time bioluminescent and fluorescent imaging

For PMT data, peaks and troughs were identified from the raw bioluminescence in wild-type oscillations. For CRY1,2-null experiments, PMT data was detrended by subtracting a polynomial fit before an FFT-NLLS fit was made in BioDARE2 (15) (https://biodare2.ed.ac.uk/) in order to assess circadian properties. Rhythmicity index was calculated by taking the mean of the autocorrelation of the detrended time series at 26, 52 and 78h, a cycle length appropriate for CRY1-driven oscillations. The autocorrelation was calculated in R using the acf command in the base R stats package.

All manipulation of real-time bioluminescence and fluorescence images was carried out in FIJI (16). Aggregate bioluminescence and fluorescence were analysed by exporting sequential images to TIFFs. Bioluminescence stacks were then de-noised by removing outliers and the raw mean grey values through the stacks were exported. For fluorescence, stacks were background subtracted by applying the built-in background subtraction command, using the rolling ball algorithm and setting the size to 5 pixels before the raw mean grey values were exported. Exported data was further analysed in Excel and R and detrended by subtracting a polynomial fit in order to identify peaks and troughs and rhythmicity indexes. Under some circumstances, peak phases, periods and relative amplitude error was identified using FFT-NLLS measures in BioDARE2 as above. Rhythmicity index was calculated as above, using the autocorrelation at 24, 48 or 72h for wild-type oscillations.

Rayleigh statistics were calculated using the r.test command in the CircStats package in R (version 0.2-6). To compare the phases of the astrocyte-initiated $[GABA]_e$ peak and trough with the wild-type phases, a modified version of the Rayleigh test was used where the reported phase was tested against an alternative hypothesis where the wild-type peak and trough timings were supplied as specified mean directions (in radians) using the v0.test command in the CircStats package.

For $GRAB_{VIP1.0}$ recordings in VIP-WT and VIP-KO SCN, jRCaMP1a and $GRAB_{VIP1.0}$ data were detrended before being normalised and overlaid as 24h intervals centred around the peak of the jRCaMP1a signal.

In order to assess the acute changes in $[GABA]_e$ dynamics in response to GAT3 inhibitor treatment, the peaks and troughs were projected forward by fitting a line to the pre-treatment peaks or troughs for vehicle and inhibitor treatment in the raw aggregate trace. This allowed prediction of the level at which these parameters would have been to allow comparison with the recorded values, which was expressed as a ratio of the actual amplitude/predicted amplitude.

Analysis of previously published scRNA-seq datasets

Data from (17) were accessed from NCBI Gene Expression Omnibus with the accession number: GSE167927. Data were analysed in R using the Seurat package (Seurat version 4.0.5, (18)). A script of the analysis is available in supplementary information.

Experimental design and statistical analysis

Where possible, slices received paired treatments (vehicle and drug) and were exposed to all concentrations of drug along a dose-response curve. Where this was not possible, or slices died during the course of the experiment, slices were assigned randomly to groups. All data were analyzed in Excel (Microsoft), R (version 3.6.1; R Foundation for Statistical Computing), RStudio (version 1.2.1335, RStudio Inc.) and GraphPad Prism 9 (GraphPad). All the statistical tests used are listed in the text and figure legends. All numbers reported in text are mean±SEM unless otherwise stated.

Supporting Information: Seurat single cell analysis script

```
#Coarsely combine, cluster and identify by time and cell-type (neurons,
astrocytes, everything else)
#day-night SCN slice data from Morris et al, 2021 EMBO J to query gene
expression
#Version 1.1 written by Andrew Patton, 10th August 2022

#Load required packages

library(Seurat)
library(dplyr)
library(patchwork)
library(hdf5r)
library(ggplot2)

#Import raw data downloaded from accession database

mmSCN5.data <-
Read10X_h5('GSM5115763_mmSCN5_filtered_gene_bc_matrices_h5.h5')
mmSCN10.data <-
Read10X_h5('GSM5115764_mmSCN10_filtered_gene_bc_matrices_h5.h5')
mmSCN6.data <-
Read10X_h5('GSM5115760_mmSCN6_filtered_gene_bc_matrices_h5.h5')
mmSCN7.data <-
Read10X_h5('GSM5115761_mmSCN7_filtered_gene_bc_matrices_h5.h5')
mmSCN11.data <-
Read10X_h5('GSM5115762_mmSCN11_filtered_gene_bc_matrices_h5.h5')

#Create seurat objects from raw data

mmSCN5 <- CreateSeuratObject(counts = mmSCN5.data, project = "mmSCN5",
min.cells = 3, min.features = 200)
mmSCN10 <- CreateSeuratObject(counts = mmSCN10.data, project =
"mmSCN10", min.cells = 3, min.features = 200)
mmSCN6 <- CreateSeuratObject(counts = mmSCN6.data, project = "mmSCN6",
min.cells = 3, min.features = 200)
mmSCN7 <- CreateSeuratObject(counts = mmSCN7.data, project = "mmSCN7",
min.cells = 3, min.features = 200)
mmSCN11 <- CreateSeuratObject(counts = mmSCN11.data, project =
"mmSCN11", min.cells = 3, min.features = 200)

#Merge datasets irrespective of time of day for cell-type clustering
```

```
All <- merge(mmSCN5, y = c(mmSCN10, mmSCN6, mmSCN7, mmSCN11),
add.cell.ids = c("SCN5", "SCN10", "SCN6", "SCN7", "SCN11"), project =
"All")
All
rm(mmSCN5.data, mmSCN10.data, mmSCN6.data, mmSCN7.data, mmSCN11.data,
mmSCN5, mmSCN6,mmSCN7, mmSCN10, mmSCN11)
gc()

#QC to remove mitochondrial genes

All[["percent.mt"]] <- PercentageFeatureSet(All, pattern = "^mt-")
VlnPlot(All, features = c("nFeature_RNA", "nCount_RNA", "percent.mt"),
ncol = 3)
All <- subset(All, subset = nFeature_RNA > 100 & nFeature_RNA < 8000 &
percent.mt < 12.5)

#Normalise the data and run clustering algorithms

All <- NormalizeData(All, normalization.method = "LogNormalize",
scale.factor = 10000)
All <- FindVariableFeatures(All, selection.method = "vst", nfeatures =
2000)
all.genes <- rownames(All)
All <- ScaleData(All, features = all.genes)
All <- RunPCA(All, features = VariableFeatures(object = All), npcs=100)
All <- FindNeighbors(All, dims = 1:15)
All <- FindClusters(All, resolution = 0.05)
All <- RunUMAP(All, dims = 1:15)
DimPlot(All, reduction = "umap")

#Check markers and assign cluster identity coarsely to allow targetted
interrogation of neuronal or astrocytic cell groups

All.markers <- FindAllMarkers(All, only.pos = TRUE, min.pct = 0.25,
logfc.threshold = 0.25)
VlnPlot(All, features = c("Tubb3", "Slc32a1", "Celf4","Gfap",
"Aldh1l1", "Aqp4", "Ndrg2", "S100b"))

new.cluster.ids <- c("Neurons", "Neurons","Astrocytes","Other",
"Other", "Astrocytes", "Other", "Other", "Other")
names(new.cluster.ids) <- levels(All)
All <- RenameIdents(All, new.cluster.ids)
DimPlot(All, reduction = "umap", label = TRUE, pt.size = 0.5) +
NoLegend()

saveRDS(All, file = "~/RProjects/Analysis/All.rds")

#Set up new metadata to allow backtracking to cell-type clusters

All$CellType <- Idents(All)

#Switch active ident to the original idents to allow time of day
alignment

Idents(All) <- "orig.ident"
table(Idents(All))
```

```
#Subset the dataset to get daytime (CT7.5) or nighttime (CT15.5) runs

All.Day <- subset(All, ident = c("mmSCN6","mmSCN7","mmSCN11"))
Idents(All.Day) <- "CellType"

All.Night <- subset(All, ident = c("mmSCN5","mmSCN10"))
Idents(All.Night) <- "CellType"

#Reassign cluster identities to take into account time cells were
harvested

new.cluster.ids.Day <- c("Neurons CT7.5", "Astrocytes CT7.5", "Other
CT7.5")
names(new.cluster.ids.Day) <- levels(All.Day)
All.Day <- RenameIdents(All.Day, new.cluster.ids.Day)

new.cluster.ids.Night <- c("Neurons CT15.5", "Astrocytes CT15.5",
"Other CT15.5")
names(new.cluster.ids.Night) <- levels(All.Night)
All.Night <- RenameIdents(All.Night, new.cluster.ids.Night)

#Merge time-aligned data sets back together and order the cell-types

All.Time <- merge(All.Day, y= All.Night, merge.data = "TRUE", project =
"All.Time")
All.Time@active.ident <- factor(All.Time@active.ident,
levels=c("Neurons CT7.5", "Astrocytes CT7.5", "Other CT7.5", "Neurons
CT15.5", "Astrocytes CT15.5", "Other CT15.5"))

#Create dot plot to show expression of genes of interest (GATs)
alongside sanity check
#(Tubb3 [B-Tubulin, Neurons] and Aldh1l1 [Astrocytes])

DotPlot(All.Time, features = c("Slc6a1", "Slc6a13", "Slc6a11",
"Slc6a12", "Tubb3", "Aldh1l1")) + scale_color_viridis_c(option =
"magma")

saveRDS(All.Time, file = ~/RProjects/Analysis/AllTime.rds")

#Redo clustering analysis to restore order and allow further future
analyses - merge does not carry over previous clustering results

All.Time.Umap <- All.Time
All.Time.Umap$CellTypeT <- Idents(All.Time.Umap)

All.Time.Umap <- NormalizeData(All.Time.Umap, normalization.method =
"LogNormalize", scale.factor = 10000)
All.Time.Umap <- FindVariableFeatures(All.Time.Umap, selection.method =
"vst", nfeatures = 2000)

all.time.umap.genes <- rownames(All.Time.Umap)
All.Time.Umap <- ScaleData(All.Time.Umap, features =
all.time.umap.genes)
All.Time.Umap <- RunPCA(All.Time.Umap, features =
VariableFeatures(object = All.Time.Umap), npcs=100)
All.Time.Umap <- FindNeighbors(All.Time.Umap, dims = 1:15)
All.Time.Umap <- FindClusters(All.Time.Umap, resolution = 0.05)
All.Time.Umap <- RunUMAP(All.Time.Umap, dims = 1:15)
```

```
DimPlot(All.Time.Umap, reduction = "umap")

#Reassign previous clusters

Idents(All.Time.Umap) <- "CellTypeT"
saveRDS(All.Time.Umap, file = "~/RProjects/Analysis/AllTime.rds")

#Run differential expression

DvN.Astros <- FindMarkers(All.Time.Umap, ident.1 = "Astrocytes CT7.5",
ident.2 = "Astrocytes CT15.5", test.use = "wilcox")

#Recreate heatmap

DoHeatmap(All.Time.Umap, features = c("Slc6a1", "Slc6a13", "Slc6a11",
"Slc6a12", "Tubb3", "Aldh1l1")) + scale_fill_continuous(type="viridis")

#Recreate dot plot

DotPlot(All.Time.Umap, features = c("Gfap", "Aqp4", "Aldh1l1",
"Slc1a3", "Slc1a2", "Sox9", "Sox3", "Slc12a2", "Slc12a5"))
+ scale_colour_continuous(type="viridis")
+ geom_point(aes(size=pct.exp), shape = 21, colour="black", stroke=0.5)
+ guides(size=guide_legend(override.aes=list(shape=21, colour="black",
fill="white")))
```

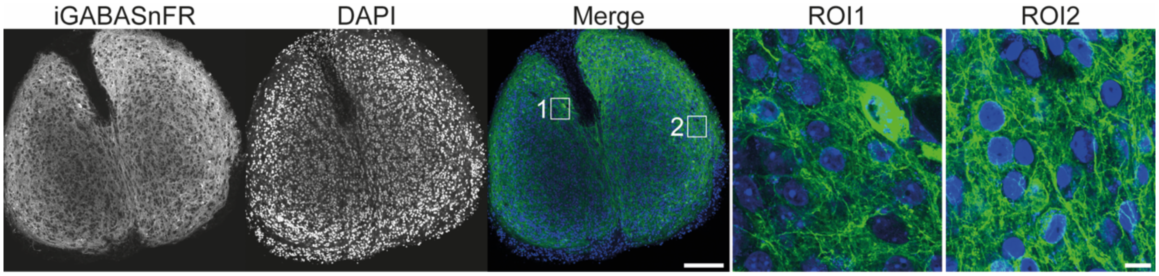

Fig. S1. iGABASnFR fluorescence is membrane targeted across the SCN. Confocal images of an SCN slice expressing neuronal-targeted iGABASnFR (left, iGABASnFR) alongside nuclear DAPI staining (middle, DAPI) and a false-coloured merged image (right, Merge, iGABASnFR (green) and DAPI (blue)). Two zoomed-in ROIs are shown to the right. Scale bar=200μm/10μm.

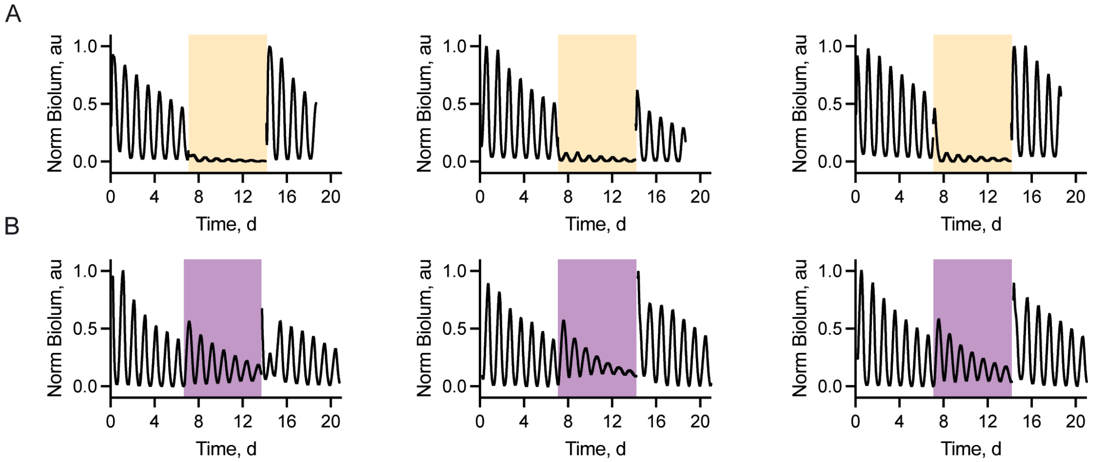

Fig. S2. The effects of chronic muscimol and (+)-bicuculline treatment are reversible on washout. A. Example normalised PMT traces showing PER2::LUC bioluminescence before, during and after treatment with 100μM muscimol (yellow). B. Example normalised PMT traces showing PER2::LUC bioluminescence before, during and after treatment with 100μM (+)-bicuculline (purple).

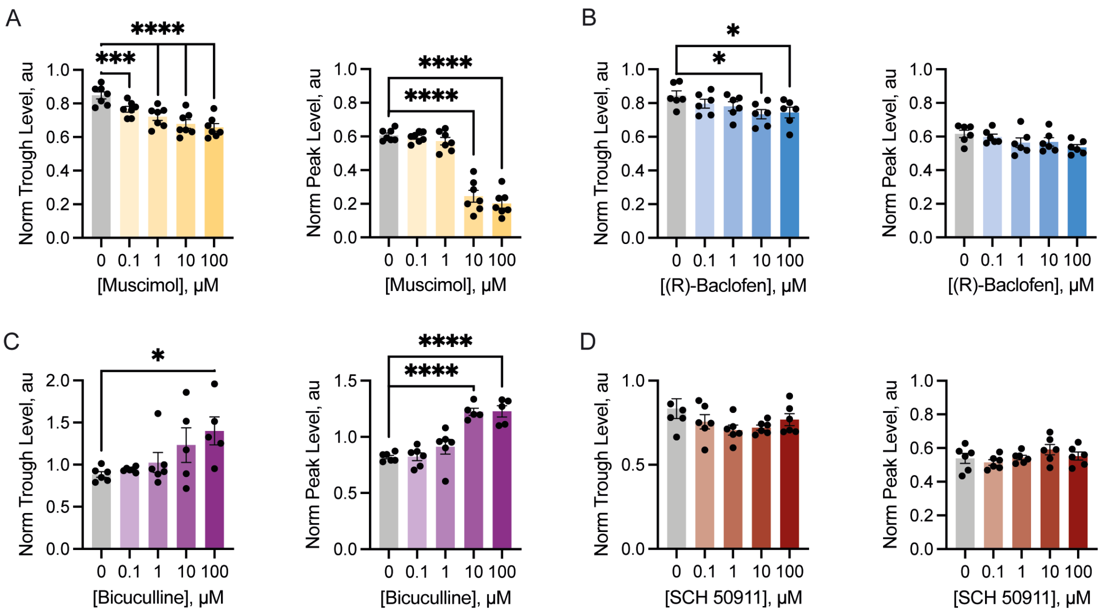

Fig. S3. Effects of chronic treatment of SCN slices with GABA$_A$ or GABA$_B$ receptor agonists and antagonists on peak and trough levels. Histograms showing PER2::LUC trough (left) or peak (right) levels of the treatment interval normalised to the baseline interval for slices treated with different concentrations of (A) muscimol, (B) (R)-baclofen, (C) (+)-bicuculline, and (D) SCH5011. In all plots, individual points represent single SCN and bars are mean±SEM.

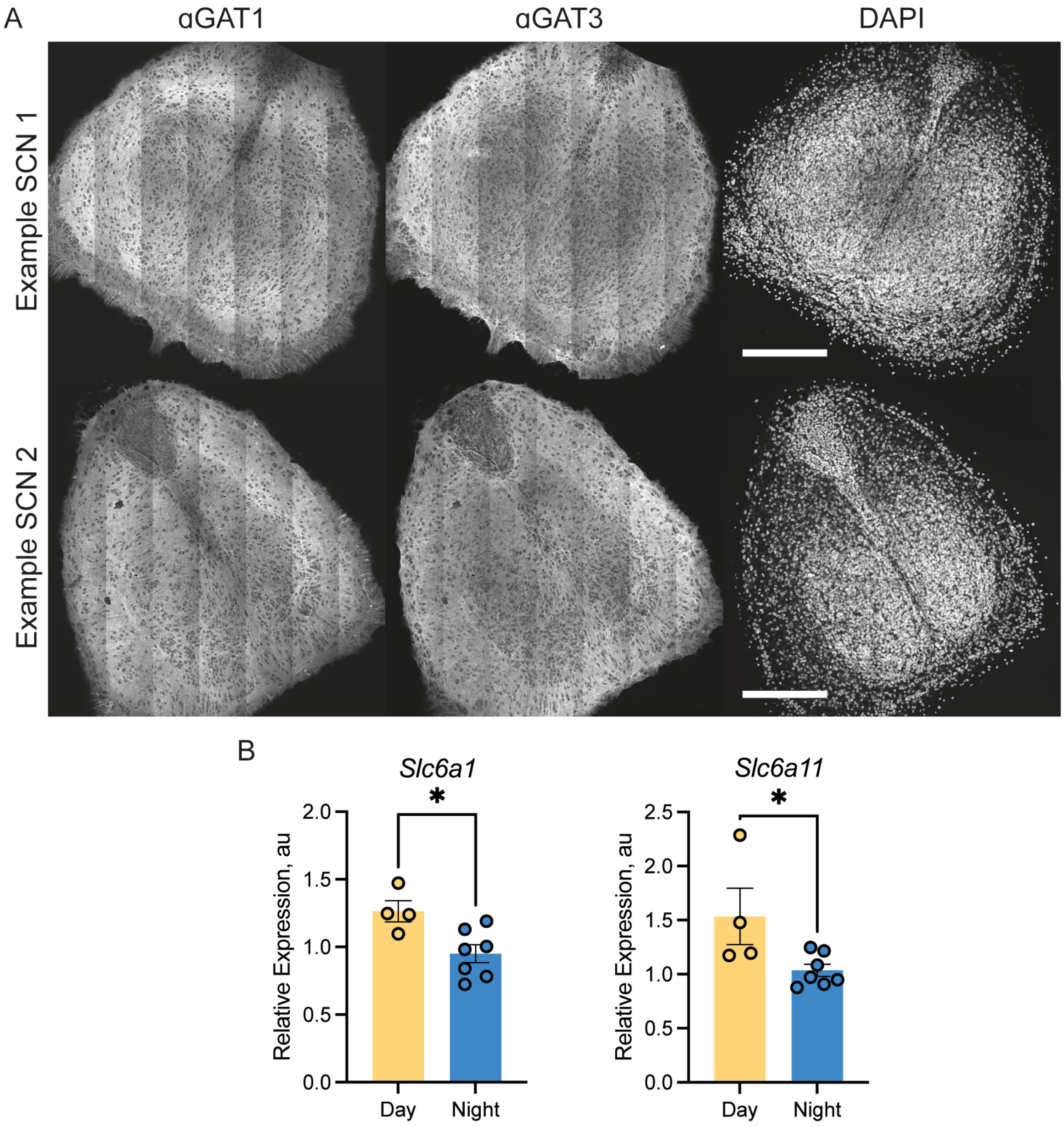

Fig. S4. GAT1 and GAT3 expression within SCN explants. Confocal micrographs showing two independent SCN explants immunostained for the GABA transporters GAT1 (left) and GAT3 (middle) alongside the nuclear stain DAPI (right). Scale bar=200μm. Explants were fixed independent of time-of-day. B. Histograms showing the relative expressions of GAT1 (*Slc6a1*, left) and GAT3 (*Slc6a11*, right) relative to the house-keeping gene *Rn18s* in slices harvested during circadian day (CT0-4) (Day, orange) or circadian night (CT12-16) (Night, blue). N=4 day/7 night. Statistics: unpaired two-tailed t-test: *Slc6a1,* t(9)=2.95, p=0.016*; Slc6a11,* t(9)=2.45, p=0.037.

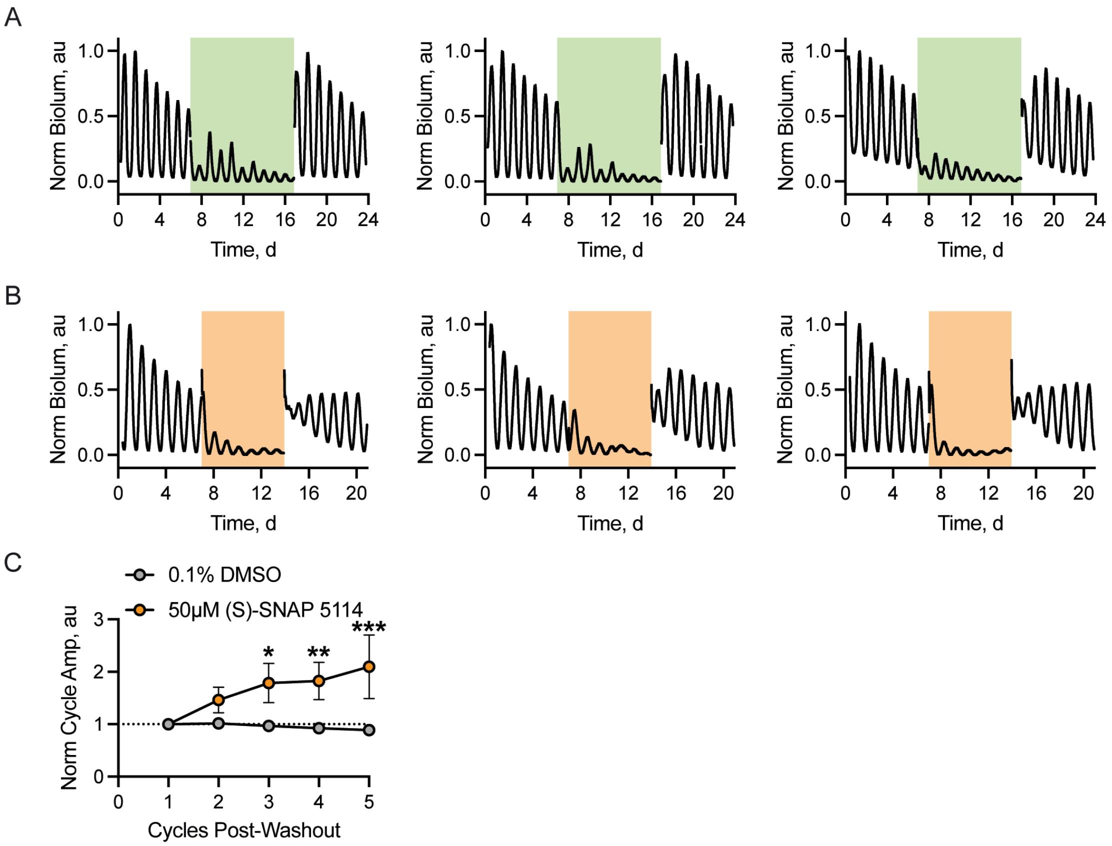

Fig. S5. The effects of CI 966 HCl and (S)-SNAP 5114 treatment are reversible upon washout. A. Example normalised PMT traces showing PER2::LUC bioluminescence before, during and after treatment with 50μM CI-966 HCl (green). B. Example normalised PMT traces showing PER2::LUC bioluminescence before, during and after treatment with 50μM (S)-SNAP 5114 (orange). C. Normalised cycle-to-cycle amplitude of the PER2::LUC oscillation post-washout for slices treated with DMSO vehicle or 50μM (S)-SNAP 5114. N=6 vehicle/4 (S)-SNAP 5114. Two-way ANOVA: Time effect: $F(4,31)=5.74$, $p=0.001$; Treatment effect: $F(1,8)=8.85$, $p=0.018$; Time x Treatment interaction: $F(4,31)=8.72$, $p<0.0001$.

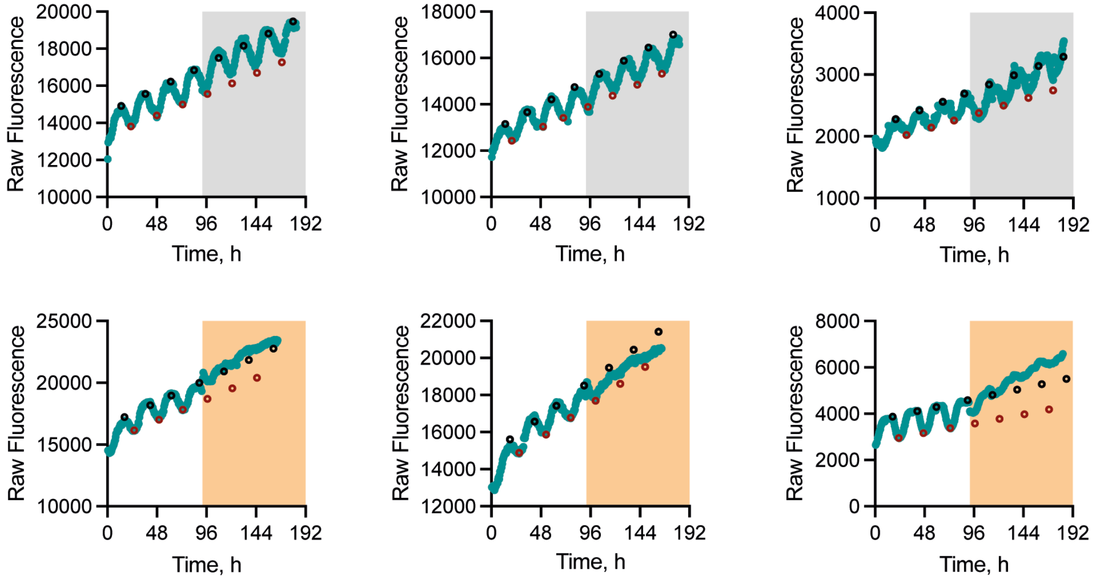

Fig. S6. Observed and extrapolated peaks and troughs in iGABASnFR-rhythms under treatment with vehicle or (S)-SNAP 5114. Example traces of raw fluorescence for slices treated with vehicle (upper, grey) or 50µM (S)-SNAP5114 (lower, orange). Predicted peak positions based on linear projections made from the baseline interval are shown as black circles, while predicted troughs are shown as maroon circles.

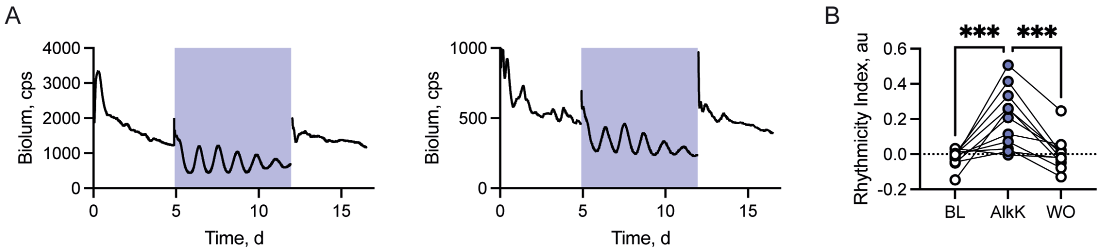

Fig. S7. AlkK treatments that initiate rhythmicity in CRY1,2-null SCN are reversible. A. Example traces of PER2::LUC bioluminescence from CRY1,2-null slices pre-treatment, during treatment and post-treatment with 10mM AlkK. Treatment interval is indicated by purple shading. B. Rhythmicity index calculated for baseline (BL), 10mM AlkK treatment (AlkK) and washout (WO) intervals. For B: N=11. Statistics: repeated-measures one-way ANOVA, $F(2,20)=15.5$, $p<0.0001$, Tukey's multiple comparisons test: ***$p<0.0006$ (comparison between BL and WO is non-significant, $p=0.88$).

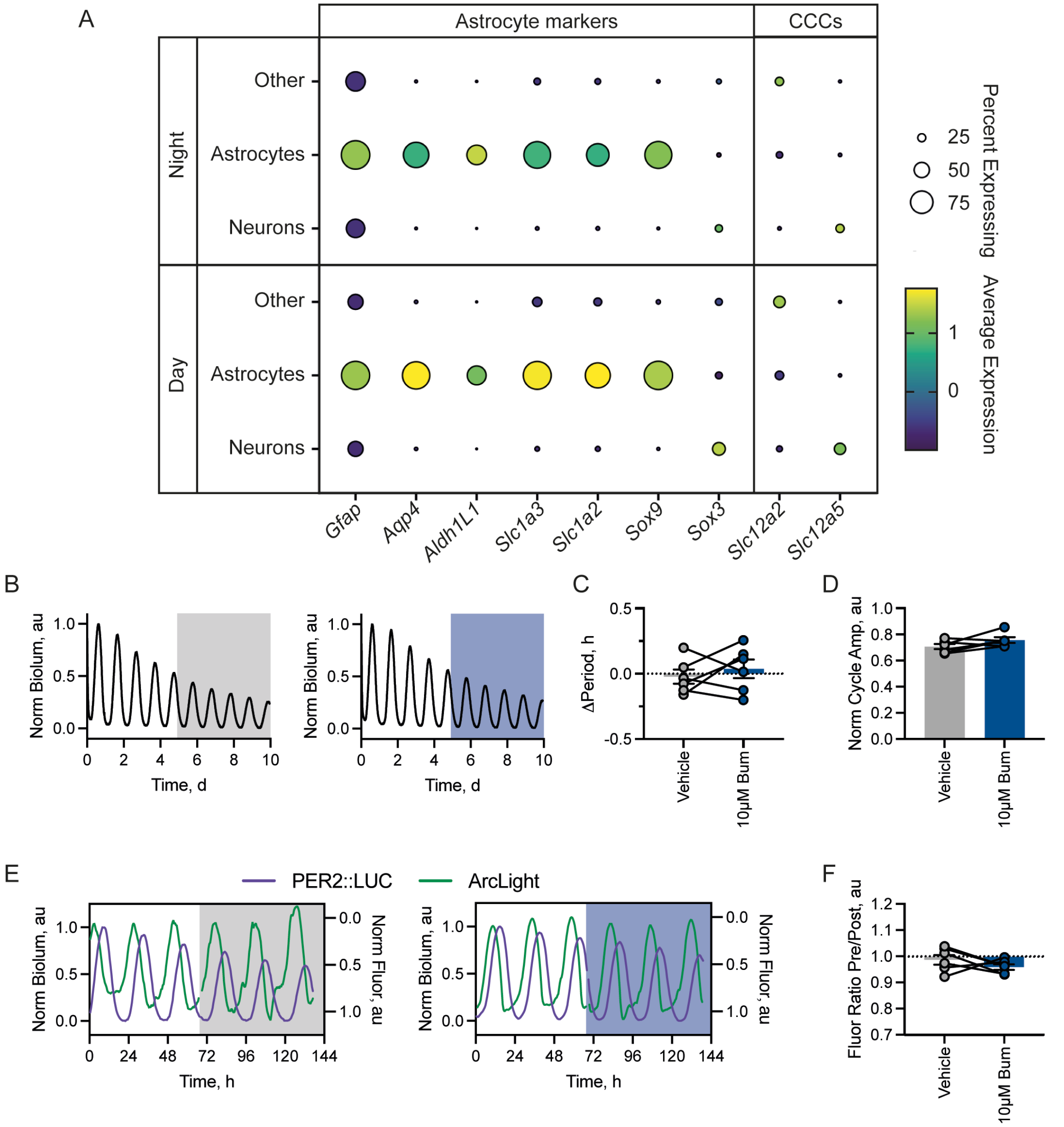

Fig. S8. Cells comprising the SCN network are mature, and their activity is not altered by NKCC1 inhibition. A. Dot plot showing the expression of mature astrocytic markers and cation chloride co-transporters (CCCs) within scRNAseq data (17) from cultured P10-12 SCN explants harvested after 3 days in culture. Dot size indicates the proportion of cells expressing that transcript and colouration shows the average expression level. Note that *Sox9* is expressed in SCN astrocytes in the absence of *Sox3*, the presence of which is an indicator of pre-developmental astrocytes (19). B. Example PMT traces from SCN explants treated with either vehicle (grey, left) or 10μM bumetanide (an NKCC1 inhibitor) (dark blue, right). C. Histogram showing the change in period between the baseline and treatment intervals for vehicle (grey) or 10μM bumetanide (dark blue) treated slices. Statistics: paired two-tailed t-test $t(5)=0.7288$, $p=0.50$. D. Histogram showing normalised cycle amplitude for vehicle (grey) or 10μM bumetanide (dark blue) treated slices. Statistics: paired two-tailed t-test $t(5)=1.875$, $p=0.12$. E. Example normalised PER2::LUC bioluminescence and *Syn*-ArcLight fluorescence from slices treated with vehicle (left, grey shading) or 10μM bumetanide (right, dark blue shading). Note: ArcLight recordings are inverted so that depolarisation and hyperpolarisation are represented by upward and downward inflections,

respectively (7). F. Relative shifts in total fluorescence expressed as a ratio of the changes over the last 24h preceding and the first 24h of treatment for vehicle (grey) and 10μM bumetanide treatment (dark blue). Statistics: paired two-tailed t-test $t(5)=1.091$, $p=0.33$. In all plots, individual points represent single SCN and bars are mean±SEM. Points are joined to represent paired measures.

Supplementary Movie Legends

Movie S1. Example recording of PER2::LUC and *Syn*.iGABASnFR (related to Fig. 1). Example video of an *ex vivo* SCN explant showing bioluminescent PER2::LUC emissions (left) alongside *Syn*.iGABASnFR fluorescence (right).

Movie S2. Vehicle treated recpording of PER2::LUC, *Syn*.jRCaMP1a and *Syn*.iGABASnFR (related to Fig. 3). Example video of an *ex vivo* SCN explant showing bioluminescent PER2::LUC emissions (left) alongside *Syn*.jRCaMP1a (middle) and *Syn*.iGABASnFR (right) fluorescence. An initial baseline interval is shown, followed by vehicle treatment as denoted in the upper left corner (+ Vehicle).

Movie S3. (S)-SNAP 5114 treated recording of PER2::LUC, Syn.jRCaMP1a and Syn.iGABASnFR (related to Fig. 3). Example video of an *ex vivo* SCN explant showing bioluminescent PER2::LUC emissions (left) alongside *Syn*.jRCaMP1a (middle) and *Syn*.iGABASnFR (right) fluorescence. An initial baseline interval is shown, followed by 50μM (S)-SNAP 5114 treatment as denoted in the upper left corner (+ 50μM (S)-SNAP5114).

Movie S4. Vehicle treated CRY-null recording of PER2::LUC and *Syn*.iGABASnFR (related to Fig. 5). Example video of a vehicle treated *ex vivo* CRY-null SCN explant expressing translationally switchable *pCry1*.CRY1::mRuby3 and astrocytically-targeted tRNA synthetase showing bioluminescent PER2::LUC emissions (left) alongside *Syn*.iGABASnFR (right) fluorescence. Vehicle was added to the medium immediately before recording started.

Movie S5. AlkK treated CRY-null recording of PER2::LUC and *Syn*.iGABASnFR (related to Fig. 5). Example video of an AlkK treated *ex vivo* CRY-null SCN explant expressing translationally switchable *pCry1*.CRY1::mRuby3 and astrocytically-targeted tRNA synthetase showing bioluminescent PER2::LUC emissions (left) alongside *Syn*.iGABASnFR (right) fluorescence. 10mM AlkK was added to the medium immediately before recording started.

SI References

1. S. H. Yoo *et al.*, PERIOD2::LUCIFERASE real-time reporting of circadian dynamics reveals persistent circadian oscillations in mouse peripheral tissues. *Proc Natl Acad Sci U S A* **101**, 5339-5346 (2004).
2. C. S. Colwell *et al.*, Disrupted circadian rhythms in VIP- and PHI-deficient mice. *Am J Physiol Regul Integr Comp Physiol* **285**, R939-949 (2003).
3. G. T. van der Horst *et al.*, Mammalian Cry1 and Cry2 are essential for maintenance of circadian rhythms. *Nature* **398**, 627-630 (1999).
4. M. H. Hastings, A. B. Reddy, D. G. McMahon, E. S. Maywood, Analysis of circadian mechanisms in the suprachiasmatic nucleus by transgenesis and biolistic transfection. *Methods Enzymol* **393**, 579-592 (2005).
5. J. S. Marvin *et al.*, A genetically encoded fluorescent sensor for in vivo imaging of GABA. *Nat Methods* **16**, 763-770 (2019).
6. H. Dana *et al.*, Sensitive red protein calcium indicators for imaging neural activity. *Elife* **5** (2016).
7. L. Jin *et al.*, Single action potentials and subthreshold electrical events imaged in neurons with a fluorescent protein voltage probe. *Neuron* **75**, 779-785 (2012).
8. H. Wang *et al.*, A toolkit of highly selective and sensitive genetically encoded neuropeptide sensors. *bioRxiv* https://doi.org/10.1101/2022.03.26.485911 (2022).
9. N. J. Smyllie *et al.*, Cryptochrome proteins regulate the circadian intracellular behavior and localization of PER2 in mouse suprachiasmatic nucleus neurons. *Proc Natl Acad Sci U S A* **119** (2022).
10. T. P. Krogager *et al.*, Labeling and identifying cell-specific proteomes in the mouse brain. *Nat Biotechnol* **36**, 156-159 (2018).
11. E. S. Maywood *et al.*, Translational switching of Cry1 protein expression confers reversible control of circadian behavior in arrhythmic Cry-deficient mice. *Proc Natl Acad Sci U S A* **115**, E12388-E12397 (2018).
12. D. McManus *et al.*, Cryptochrome 1 as a state variable of the circadian clockwork of the suprachiasmatic nucleus: Evidence from translational switching. *Proc Natl Acad Sci U S A* **119**, e2203563119 (2022).
13. F. P. David, J. Rougemont, B. Deplancke, GETPrime 2.0: gene- and transcript-specific qPCR primers for 13 species including polymorphisms. *Nucleic Acids Res* **45**, D56-D60 (2017).
14. M. W. Pfaffl, A new mathematical model for relative quantification in real-time RT-PCR. *Nucleic Acids Res* **29**, e45 (2001).
15. T. Zielinski, A. M. Moore, E. Troup, K. J. Halliday, A. J. Millar, Strengths and limitations of period estimation methods for circadian data. *PLoS One* **9**, e96462 (2014).
16. J. Schindelin *et al.*, Fiji: an open-source platform for biological-image analysis. *Nat Methods* **9**, 676-682 (2012).
17. E. L. Morris *et al.*, Single-cell transcriptomics of suprachiasmatic nuclei reveal a Prokineticin-driven circadian network. *EMBO J* **40**, e108614 (2021).
18. Y. Hao *et al.*, Integrated analysis of multimodal single-cell data. *Cell* **184**, 3573-3587 e3529 (2021).
19. S. Klum *et al.*, Sequentially acting SOX proteins orchestrate astrocyte- and oligodendrocyte-specific gene expression. *EMBO Rep* **19** (2018).
